# Isolation methods and characterization of primary rat neurovascular cells

**DOI:** 10.1186/s13036-024-00434-3

**Published:** 2024-07-11

**Authors:** Sydney Floryanzia, Seoyoung Lee, Elizabeth Nance

**Affiliations:** 1 Department of Chemical Engineering, University of Washington, Seattle, WA 98195 USA; 2 Department of Bioengineering, University of Washington, Seattle, WA 98195 USA; 3 Department of Molecular Engineering and Sciences, University of Washington, Seattle, WA 98195 USA

**Keywords:** Primary blood-brain barrier cell isolation, Astrocytes, Endothelial cells, Pericytes

## Abstract

**Background:**

There is significant interest in isolating cells of the blood-brain barrier (BBB) for use in in vitro screening of therapeutics and analyzing cell specific roles in neurovascular pathology. Primary brain cells play an advantageous role in BBB models; however, isolation procedures often do not produce cells at high enough yields for experiments. In addition, although numerous reports provide primary cell isolation methods, the field is lacking in documentation and detail of expected morphological changes that occur throughout culturing and there are minimal troubleshooting resources. Here, we present simplified, robust, and reproducible methodology for isolating astrocytes, pericytes, and endothelial cells, and demonstrate several morphological benchmarks for each cell type throughout the process and culture timeframe. We also analyze common considerations for developing neurovascular cell isolation procedures and recommend solutions for troubleshooting.

**Results:**

The presented methodology isolated astrocytes, pericytes, and endothelial cells and enabled cell attachment, maturation, and cell viability. We characterized milestones in cell maturation over 12 days in culture, a common timeline for applications of these cell types in BBB models. Phase contrast microscopy was used to show initial cell plating, attachment, and daily growth of isolated cells. Confocal microscopy images were analyzed to determine the identity of cell types and changes to cell morphology. Nuclear staining was also used to show the viability and proliferation of glial cells at four time points. Astrocyte branches became numerous and complex with increased culture time. Microglia, oligodendrocytes, and neurons were present in mixed glial cultures for 12 days, though the percentage of microglia and neurons expectedly decreased after passaging, with microglia demonstrating a less branched morphology.

**Conclusions:**

Neurovascular cells can be isolated through our optimized protocols that minimize cell loss and encourage the adhesion and proliferation of isolated cells. By identifying timepoints of viable glia and neurons within an astrocyte-dominant mixed culture, these cells can be used to evaluate drug targeting, uptake studies, and response to pathological stimulus in the neurovascular unit.

**Supplementary Information:**

The online version contains supplementary material available at 10.1186/s13036-024-00434-3.

## Background

The blood-brain barrier (BBB) protects the brain parenchyma from toxic molecules in the blood and also presents a challenge for drug delivery into the brain [1]. There is thus significant interest in utilizing in vitro models of the BBB for screening of compounds that may drive disease or be therapeutically relevant. Primary cells derived from whole brain tissue can be useful in these models to include regional differences in cells as well as the ability to isolate different cell types at once. Previous work has demonstrated the utility of human [2], murine [3, 4], porcine [5], and other animal brain tissue sources in isolating astrocytes, pericytes, and endothelial cells for use in vitro. However, to maximize the advantages that primary cells offer in BBB studies, continued efforts are needed to harmonize established protocols, identify best practices, and develop simplified, effective methods that produce high overall cell yields.

### Cells of the BBB and neurovascular unit

In vitro models of the BBB commonly include astrocytes, pericytes, and endothelial cells, as these cell types are the main cellular components of this barrier. Astrocytes contribute to homeostasis of the brain microenvironment through a variety of functions [6] such as physical support for the BBB, metabolic regulation and debris clearance with some phagocytic activity [7], and transport of water, ions, amino acids, and neurotransmitters. Pericytes are thought to play a beneficial role in clearance of toxic molecules, provide support for the BBB, and regulate blood flow by modulating capillary diameter to participate with astrocytes in neurovascular coupling [8–10]. Endothelial cells share the basement membrane with pericytes and line the inside of vasculature to directly interface with the blood. The lumen of capillaries is created from one or two endothelial cells wrapping around one another [9, 11]. Endothelial cells produce tight junction proteins between adjacent cells that limit paracellular permeability, have few vesicles which limits transcytosis, and have efflux pumps that together contribute to the restrictive properties of the BBB [10].

Endothelial cell tight junction proteins can be regulated by pericytes and influenced by astrocytes through molecular secretions such as Sonic hedgehog (Shh) [10, 11]. Astrocytes communicate directly with neurons and link them to the BBB and thus the rest of the body [6, 12]. Neurons can also influence astrocyte secretion which in turn influences the BBB. Microglia, often described as the brain’s resident immune cells [10], participate in phagocytotic clearance and engage in crosstalk with astrocytes and oligodendrocytes. The resulting cascade of molecular signaling can affect endothelial cells through the release of cytokines and other molecules that can worsen disruption of the BBB or repair its function. The constant and intricate interactions of neuron-glia, including microglia and oligodendrocytes, and the BBB are collectively referred to as the neurovascular unit (NVU) [8, 10, 12] and contribute to the immune and inflammatory responses of the brain [7, 13]. The direct influences of the NVU on barrier strength both in healthy and pathological conditions can in turn affect BBB interactions with therapeutic molecules. Thus, it can be helpful for in vitro models to expand the cell types traditionally included – beyond astrocytes, pericytes, and endothelial cells – to account for crosstalk between cells that could affect barrier function, drug delivery, cellular response, and therapeutic outcomes.

### BBB cell morphology and role in disease progression

Distinct cell identity, function, and effects of crosstalk can be reflected in cell morphology. While all astrocytes have a stellate morphology in general, white matter astrocytes are often described as having a fibrous morphology characterized by long processes with fewer branches and higher glial fibrillary acidic protein (GFAP) expression [14]. Gray matter astrocytes are described as having a protoplasmic morphology characterized by shorter, wider, processes with more branches. Astrocyte morphology is also frequently used to specifically indicate reactive astrogliosis, a neuroinflammatory response in which astrocytes adopt a highly ramified and hypertrophic phenotype including wider somas, numerous thick branches, and increased GFAP expression [14, 15]. Reactive astrogliosis has been implicated in several pathological conditions including ischemia [16], neurodegenerative diseases [17], and trauma, among others [15, 18, 19].

Microglia are also ramified cells with branches that dynamically change and enable diverse functions in different disease states. When branched, microglia express low cytokine levels and contribute to neuronal function. In pathological states, microglia shift from a branched phenotype to amoeboid shapes corresponding with increased migration and phagocytic activity [16, 19]. Microglia are also highly responsive to activated astrocytes, damaged neurons, and chondroitin sulfate proteoglycan releasing oligodendrocytes which can lead to astrogliosis and overall amplifying of adverse pathological effects [9, 20].

Pericyte morphology has been used in part to assist in differentiating pericytes from smooth muscle cells. In vivo, pericytes exhibit “bump on a log” morphology reflecting their nuclei appearing as “bumps” protruding from the rest of the cell wrapped tightly around small vessels [21, 22]. Pericyte morphology can also indicate cell maturity and dysfunction. The wide, rhomboid projections and thin finger-like edges of pericytes allow them to effectively ensheath capillaries and contribute to the BBB. Emerging work has shown retention of this morphology in primary cultured pericytes that can be further categorized as standard, circular, sheet, spindle, or balling based on their projections, nuclei, surface area, distribution of lamellipodial sheets, and overall shape [23]. Just as seen with astrocytes and microglia, pericytes also exhibit morphology shifts, namely constriction, in response to disease leading to increased BBB permeability.

Endothelial cell branching does not change in the presence of stimuli or disease, however it is important to note that endothelial cells in the BBB specifically can be differentiated from endothelial cells from other tissues partially through phenomic analysis. Brain microvascular endothelial cells have ovular nuclei, no additional projections, a narrow width and more elongated somas that grow tightly packed alongside one another as long spindles packed in cobblestone-like patterns. The ability for cells to replicate in vivo morphology and function – and thus their utility in in vitro BBB models – largely depends on the cell source and culture conditions.

### BBB cell sources

Cells used for vitro therapeutic screening platforms can be sourced from immortalized lines, induced pluripotent stem cells (iPSCs), or isolated from primary tissue. The growth process and culture techniques for immortalized cell lines are well-established and characterized in literature with little variability, yet the clinical relevance of immortalized cells is limited due to loss of some cell function when culturing over extended generations [24, 25]. iPSCs offer an alternative due to the ability to differentiate into virtually any cell type and the emerging use of patient-derived cells to gain patient-specific results [25, 26]. In contrast with immortalized cells, obtaining iPSCs requires complex culture conditions with high potential for variability [27], and differentiation into unintended cell phenotypes can be unavoidable [25]. Primary cells offer a balance in both the benefits and downsides of immortalized cells and iPSCs. Primary neurovascular cells used in low passage numbers retain much of their in vivo function [15, 28], including barrier properties [24, 25], and can be more directly compared to in vivo animal studies [3]. Additionally, regional, age, and sex-based differences in primary cells in response to stimuli can be ascertained [1]. However, primary cell isolations often yield lower numbers of cells and require access to live tissue [25, 26]. Lack of clarity, specificity, and consistency in many aspects of primary cell isolation procedures, including required media components, enzyme concentrations and combinations, misidentification of isopycnic separation layers, and laborious processes with low yields, have led to poor outcomes with primary cell usage and limited adoption by many labs [23, 28].

Despite these drawbacks, several studies that have used primary cells have produced effective and clinically-relevant in vitro BBB models as determined through trans-epithelial electrical resistance (TEER) or permeability assays [1, 4, 29–31]. While many protocols share a similar foundation, they diverge in important methodological steps that can be contradictory from protocol to protocol and negatively impact reproducibility; including disparities that impact outcomes obtained in BBB model testing. Isolation procedures, culture conditions, animal source, number and age differences significantly affect primary neurovascular cell function, purity, and yield [14, 32, 33]. Of the numerous existing protocols for the isolation of astrocytes, pericytes, and endothelial cells from murine brains, most do not provide a sufficient temporal morphological guide of cell attachment, growth, and maturation following isolation nor explanation for selection of reagents or isolation techniques that are needed to establish standardized approaches or compare isolation efficiency. Here, we provide robust and reproducible methodology and morphological characterization of isolated primary astrocyte, pericyte, and endothelial cells as well as comparative analysis to existing protocols, using assessment tools that are also broadly accessible to any lab. In our results and discussion, we identify critical process steps and troubleshooting strategies, then present opportunities for future research enabled by the standardized methodology and morphological characterization established in the presented work.

## Methods

### Chemicals and materials

High glucose Dulbecco’s Modified Eagle Medium (DMEM,11,965,092), low glucose DMEM (11,885,084), DMEM/Nutrient Mixture F-12 (DMEM/F12, 11,320,033), Hanks’ Balanced Salt Solution (HBSS, 14,185,052), Trypsin-EDTA (0.25%, 25,200,056), penicillin-streptomycin (10,000 U/mL, 15,140,122), and collagenase type II powder (17-101-015) were purchased through Gibco. Gentamycin sulfate (345,814-M), D-(+)-Glucose (G7021), basic fibroblast growth factor (bFGF, GF003AF), heparin (H3393), poly-L-lysine solution (PLL, P4707), fibronectin (F0895), collagen type IV (C6745), and collagen 1 from rat tail (C7661) were obtained from Sigma-Aldrich. Phosphate Buffered Saline (PBS) dry-blend buffered packs (28,372) and puromycin (AAJ67236XF) were purchased through Thermo Scientific. Fisher BioReagents™ Bovine Serum Albumin (Fraction V) Heat Shock Treated (BP1600-100) was obtained through Fisher Scientific and Percoll™ Centrifugation Media (17,089,101) was obtained through Cytiva. Insulin-transferrin-sodium selenite supplement (11,074,547,001) and collagenase/dispase^®^ (10,269,638,001) were purchased through Roche. Fetal Bovine Serum (FBS, S12450H) was acquired from R&D Systems (Formerly Atlanta Biologics), platelet-poor bovine plasma-derived serum (BPDS) was purchased from Animal Technologies, Inc, and Falcon™ 70-micron Cell Strainers (08-771-2) were obtained from Corning™. Pentobarbital was obtained from Commercial Beuthanasia D. Anti-chicken glial fibrillary acidic protein (GFAP, ab134436), anti-rabbit oligodendrocyte transcription factor (Olig2, ab109186), anti-rabbit neural/glial antigen 2 (NG2, ab275024), and anti-mouse microtubule-associated protein 2 (MAP2, ab11268), were obtained through Abcam. Anti-rabbit zonula occludens (ZO-1, 61-7300) and DAPI (4′,6-diamidino-2-phenylindole, D1306) were purchased through Invitrogen while anti-rabbit ionized calcium binding adaptor molecule 1 (Iba1, 019-19741) was obtained through Wako.

### General isolation procedures

The aim of this study was to isolate primary neurovascular cells from whole brains according to isolation techniques that enrich for specific cell types such as astrocytes, pericytes, and endothelial cells. Here, brain tissue was obtained from neonatal Sprague-Dawley rats following euthanasia sacrifice via overdose with pentobarbital and CO_2_ exposure. Isolation methods that remain consistent between obtaining astrocyte, pericyte, and endothelial cultures are described below. Seeding, passaging, and plating ratios for each cell type are summarized in Table 1.

#### Tissue extraction

**Fig. 1:**
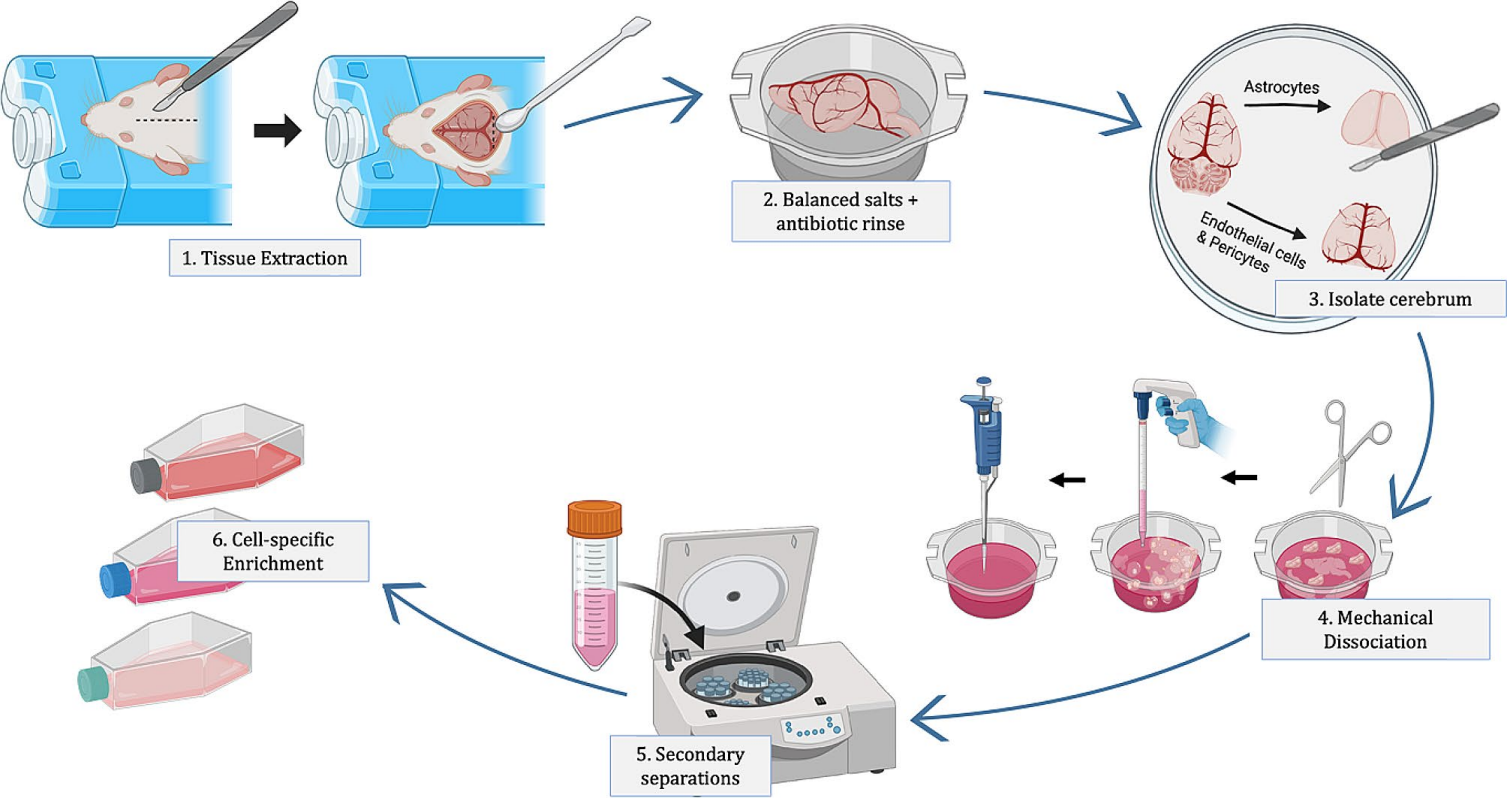
General overview for primary neurovascular cell isolation. The isolation procedure for astrocytes, pericytes, and endothelial cells, generally follows a similar sequence of five steps: (1) Tissue Extraction; starting with extracting the tissue following euthanasia, (2) Antibiotic Rinse; an introduction to antibiotic media, (3) Cerebral Isolation; by removing the meninges, olfactory bulb, and cerebellum, (4) Using mechanical dissociation techniques, (5) Additional Separations; to start separating specific cell types and (6) Cell enrichment; encouraging specific cell adhesion and proliferation through media components and surface coatings. Steps 1–4 produce a single cell suspension from brain tissue while Steps 5 and 6 produce specific cell types. This figure was created with Biorender.com

Whole brain extraction following sacrifice is conducted either on postnatal day 5–7 (P5-P7) to isolate astrocytes or at 8–9 weeks after birth to obtain pericytes and endothelial cells. After confirmed death, the head and neck are sprayed with 70% EtOH and the brain is extracted over ice. Making an initial incision along the midline of the head, the skull is removed starting from the cerebellum up to and not including the olfactory bulb (Fig. 1). These steps are accomplished with fine-tipped scissors for P5-P7 pups and with rongeurs for rats at 8 weeks and above. During removal of the skull and brain extraction, meninges are also peeled back and removed with forceps. Following extraction, the brain is immediately engulfed in dissection media consisting of a balanced salt and antibiotic solution comprised of HBSS supplemented with glucose and penicillin-streptomycin (P/S) as a first exposure to sterile conditions. All tissue is thereafter handled via aseptic technique within sterile biological safety cabinets.

#### Cerebrum isolation

Parts of the olfactory bulb and cerebrum still attached to the brain after extraction are removed after transition to the sterile hood. In this study, cerebral neurovascular cells were isolated to compare with findings from previous investigations within our lab using organotypic whole hemisphere coronal brain slices [34]. When isolating for astrocytes, the cerebrum is placed in a dish with dissection media over an ice block and visible surface vessels on the dorsal and ventral surfaces of the brain are manually removed to reduce potential contamination by fibroblasts or meningeal cells using fine-tipped forceps. In pericytes and endothelial cell isolations, additional separation steps were completed to remove other cell types including fibroblasts and meningeal cells.

#### Mechanical dissociation

The final common step in the isolation procedures for astrocytes, pericytes, and endothelial cells is mechanical dissociation of the cerebral tissue. The tissue is minced with fine-tipped scissors in DMEM supplemented with glucose, balanced salts, antibiotics and serum. These components protect cells and prevent additional trauma during dissociation by reducing tissue adherence to scissors. Tissue is minced until pieces are approximately 1 mm x 1 mm in size. After this, tissue is triturated using a 5- or 10-mL serological pipette for 20 gentle suspensions up and down. Tissue is then small enough to be triturated with a 1 mL micropipette for 10 gentle suspensions. It is not recommended that additional or harsh trituration be performed, as cells can lyse and not have the ability to repair or proliferate in culture.

### Glial cell isolation procedures

**Fig. 2:**
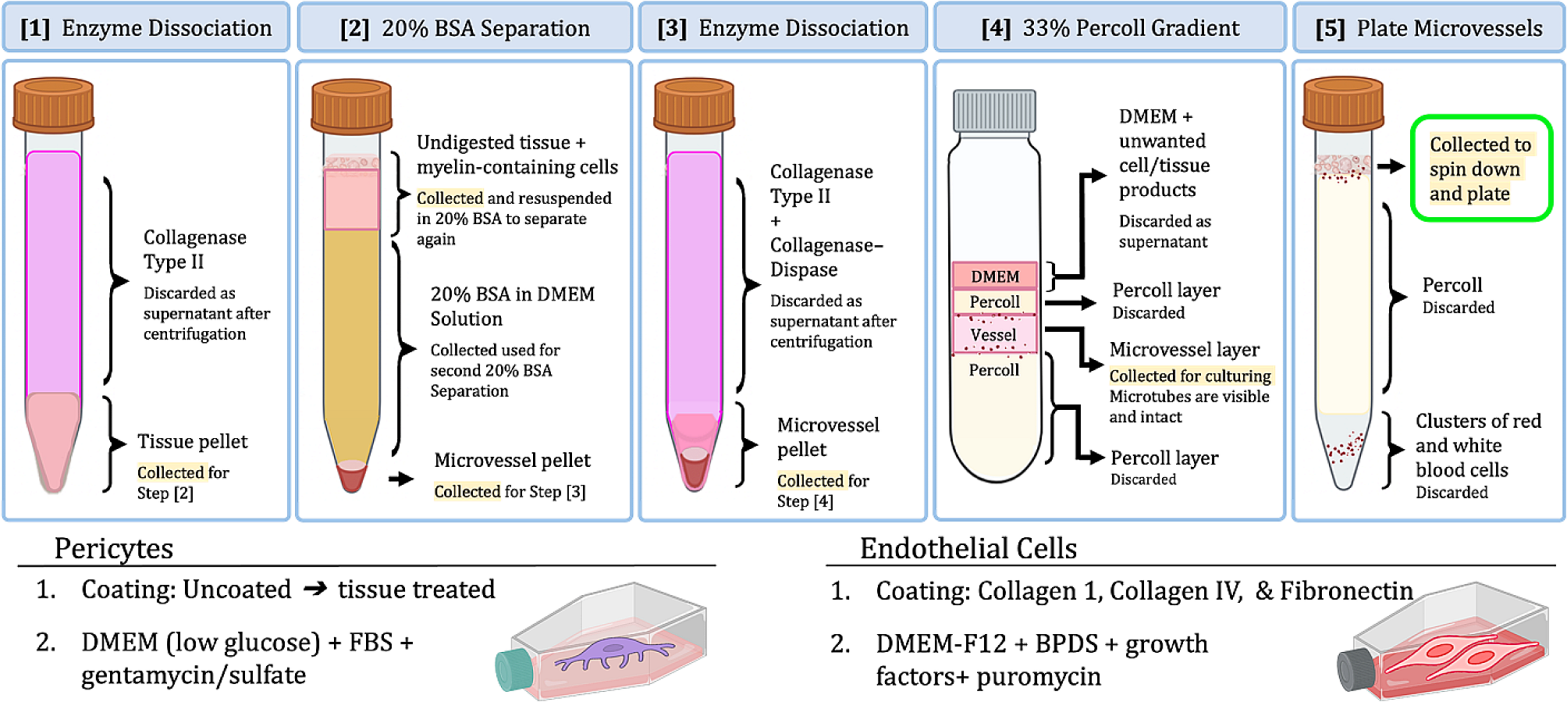
Summary of critical microvascular isolation steps. Through the microvascular isolation procedure, five critical steps greatly influence the effectiveness and yield of each cell type. Cells can be over-dissociated or digested during Step [1] which would be evidenced in Step [2]. Step [2] can be run again to ensure all vascular cells are captured. Similar risks of Step [1] exist at Step [3]. The final product of Step [3] is a mixed vascular pellet that is further separated at Step [4]. Four distinct layers with visible vascular fragments should be seen at Step [4]. The vascular layers in Step [4] are further separated at Step [5] where the top layer contains the endothelial cells and pericytes. To enrich for pericytes, plates should be uncoated. Collagen and fibronectin coatings are needed for endothelial cell attachment and proliferation. This figure was created with Biorender.com

Primary astrocytes can be derived from and enriched within mixed glial cultures. After the successive mechanical dissociation, the tissue solution is passed through a 70 μm nylon cell strainer to allow a cell suspension to pass through, while vessel fragments left behind from the surface vessel removal are captured (Fig. 2). The filtered tissue suspension is spun down using standard centrifugation (500 g, 5 min). The resulting tissue pellet is resuspended at a concentration of 0.2 mL per 1 cm^2^ of the target culture vessel growth area according to seeding ratios featured in Table 1. The desired culture vessel is coated with Poly-D-Lysine (PDL) or Poly-L-Lysine (PLL) which promote attachment of astrocytes [3] due to their positive charge and ability to bind to laminin, fibronectin, and proteins [35] that are naturally produced by astrocytes in vivo role to help to build the basement membrane of the BBB. Cells were kept in an incubator at 37 °C and 5% CO_2_ with media changes (DMEM (4 g/L D-glucose), 10% FBS, and 1% penicillin/streptomycin) preformed every 2–3 days throughout the duration of culture. Attaining a purer astrocyte culture, as opposed to an astrocyte dominant mixed culture, could be accomplished by culturing cells on an orbital shaker [1, 3] among other methods captured in Table 2. In the present study, cells were not cultured on a shaker, as one goal of the work was to co-isolate astrocytes and glial cells from the neurovascular unit. At 3 days in vitro (DIV), 5 DIV, 10 DIV, and 12 DIV, cells were fixed with 100% ice cold methanol for 10 min at -20 °C prior to imaging.

**Table 1:** Seeding, plating, and passaging ratios for primary cell isolation. Isolation components for each cell type include the resuspension volume, plating volume, and number of vessels that can be seeded for primary astrocytes, pericytes, and endothelial cells based on the size of the desired culture vessel. The number of brains used, age of donor, and passage information for each cell type are also included

**Astrocyte & Glia isolation**			For one P5-P7 brain
Vessel size	Resuspension volume	Plating volume	Number of vessels seeded
T-75 Flask	15 mL	15 mL	1
T-25 Flask	15 mL	5 mL	3
35 mm dish	15 mL	1.66 mL	9
*Passage astrocytes & glia at 5 DIV in a 1:10 split ratio*			
**Pericyte isolation**			For two P56-63 brains
Vessel size	Resuspension volume	Plating volume	Number of vessels seeded
T-75 Flask	9 mL	9 mL	1
35 mm dish	9 mL	1 mL	9
*Passage pericytes at 15 DIV in a 1:3 split ratio*			
**Endothelial cell isolation**			For two P56-63 brains
Vessel size	Resuspension volume	Plating volume	Number of vessels seeded
100 mm dish	18 mL	9 mL	2
35 mm dish	18 mL	1 mL	18
*Passage endothelial cells at 5 DIV in a 1:2 split ratio*			

**Table 2:** Procedural steps for isolation of primary astrocytes, pericytes, and endothelial cells highlights variations in details of conserved steps between many previously published primary BBB cell isolation protocols and includes references where each of the protocol details are featured

Cell type	Species and age	Reference	Step	Reference
Astrocytes	Mouse: 0 days	[36]	Remove surface vessels	[1, 3, 29, 37], [36],
	Mouse: 1–4 days	[3, 37]	Mince + pipette trituration	[1, 29–31], [38],
	Rat (SD): 0–2 days	[31]	Enzyme incubation: trypsin, 30 min, 37 °C	[3, 31],
	Rat (W): 1–3 days	[1]	Enzyme incubation: DNAse + trypsin, 20 min, 25 °C	[36]
	Rat (SD): 2 weeks	[29]	Enzyme incubation: Collagenase Type II + DNase, 10 min, 25 °C	[37]
	Pig: 6 months	[5, 30]	Enzyme incubation: Papain + DNase	[39]
			40-70-µm Cell strainer	[1, 29, 31, 39],
			Vigorous orbital shaker after 8–14 days	[1, 3, 29, 31],
			Tapping or orbital shaker at confluency	[4, 36, 38]
Pericytes & endothelial cells	Mouse: 6–10 weeks	[37, 40–45]	Euthanasia method (CO_2_ or isofluorane)	[2, 29, 31, 37, 40, 45]
	Mouse: 40–52 weeks	[40]	Roll brain on sterile gauze, blotter paper, filter paper, or cellulose chromatography paper to remove leptomeningeal cells	[4, 31, 37, 40, 41],
	Rat (W): 3 weeks	[1]	Manually remove meninges with forceps	[1, 29–31, 37, 38]
	Rat (W): 8 weeks	[1]	Mince + pipette trituration (1mm^3^ pieces)	[4, 29, 31, 37, 38, 40, 41, 45]
	Rat (SD): 2–3 weeks	[29, 30]	Mince with tissue grinder	[40]
	Rat (SD): 3 months	[20]	1st Enzymatic Digestion: DNAse 1 + TLCK + collagenase/dispase 1 h, 37 °C	[31, 40]
			1st Enzymatic Digestion: MEM + papain and DNase I, 1 h 10 min, 37 °C	[43, 45]
			1st Enzymatic Digestion: Collagenase Type II (1 mg/mL) 1.5 h, 37 °C	[1]
			1st Enzymatic Digestion: Collagenase Type II (1 mg/mL) + DNAse (15 µg/mL) 1–1.5 h, 37 °C	[4, 30, 38]
			1st Enzymatic Digestion: Collagenase Type II (1 mg/mL) + DNAse 1.25 h, 37 °C, on benchtop orbital shaker	[37, 41]
			1st Enzymatic Digestion: Collagenase/Dispase (270 U/mL and 0.1%) + DNAse (10 U/mL) 1.5 h, 37 °C	[29, 42, 43]
			18% Dextran in DMEM, vortex, add enzymes, then centrifuge 6080 x g, 10 min, 4 °C.	[40]
			20–30% BSA in DMEM, then centrifuge ~ 1000xg, 15–20 min	[1, 4, 29–31, 37, 38, 41, 43, 45]
			2nd 20–30% BSA separation	[31, 37]
			2nd Enzymatic Digestion: Collagenase/Dispase (1 mg/mL), 1 h, 37 °C,	[1, 4, 29]
			2nd Enzymatic Digestion: Collagenase/Dispase (1 mg/mL) + DNAse (6.7 µg/ml in DMEM 50 min–1 h, 37 °C	[30, 37, 38, 41]
			33% Continuous Percoll separation	[1, 4, 30, 31, 37, 38, 41–43]

*W Wistar, *SD* Sprague Dawley

### Vascular cell isolation procedures

Pericyte and endothelial cells are closely associated with vasculature and require additional isolation procedures compared to astrocytes and glial cells. Due to the proximity of pericytes and endothelial cells with one another in vivo, the isolation protocols for these cells are almost identical in methodology and primarily diverge in composition of culture media and plate coatings. Successful isolation of endothelial cells is only possible if microtubules of endothelial cells remain intact after obtaining a cell solution. The following procedural steps have been optimized to retain these microtubules and minimize cell loss due to over processing. These steps apply to the isolation of pericytes and endothelial cells which continues immediately following the general isolation procedure steps described in the General Isolation Procedures section.

#### Enzymatic dissociation

The use of enzymes can reduce the physical trauma experienced during mechanical dissociation by reducing the force required for breaking apart tissue. However, high concentrations of enzymes in solution can also over-digest tissue and degrade cell membranes in addition to breaking down connective proteins. In the present study, the tissue is triturated in a collagenase type II-DMEM solution (1 mg/mL) after mincing in DMEM alone. Collagenase type II, also known as matrix metalloproteinase 8 (MMP-8) [46], selectively cleaves type I, type II, and type III collagens present in the extracellular matrix and broad connective tissue [47]. After 20 gentle suspensions with a 5–10 mL serological pipette and 10 suspensions with the micropipette, the tissue-media-collagenase type II solution is left to incubate (50 min, 37 °C) to allow for increased enzymatic degradation. After a 20% BSA separation, discussed in the next section, the tissue is further digested through a second enzyme incubation with collagenase type II (0.5 mg/mL) in combination with collagenase/dispase (2 mg/mL) and DMEM is carried out (45 min, 37 °C). Collagenase/dispase cleaves fibronectin and type IV collagen [48], proteins found in the basement membrane on which endothelial cells align and within which pericytes are imbedded in vivo. Collagenase/dispase cleaves these bonds to release pericytes and break the basement membrane into fragments yet, at this concentration, it does not fully degrade the membrane in each fragment. Preservation of basement membrane fragments can support endothelial survival after isolation and plating by functioning as a scaffold to hold adjacent endothelial cells facilitating the retention of endothelial clusters.

#### Density-based separation steps

The pericyte and endothelial isolation procedures include two isopycnic density gradient centrifugation separations. After terminating the first enzyme reaction by centrifugation and removing the enzyme-media solution, the resulting tissue pellet is resuspended in a 20% BSA-DMEM solution and centrifuged (1000 g, 20 min, 4 °C). This results in a separation of the brain microvessel-associated cells which are retained in the pellet from the myelin-associated cells, neurons and glia, which are less dense than the BSA solution and vessels and thus appear as a distinct layer high in the conical tube (Fig. 2). The supernatant – comprised of the remaining 20% BSA solution, undigested tissue, and myelin-associating cell layers – can be collected and spun again at the same conditions to ensure all microvasculature is separated. The original pellet is left on ice while the supernatant and remaining 20% BSA-DMEM solution are centrifuged again under the same conditions. A continuous 33% Percoll gradient is run to separate vascular-associated pericytes and endothelial cells from vascular cells such as red and white blood cells. In the present study, a 33% Percoll gradient was obtained by adding 1 mL 10x PBS, 9 mL Percoll, 1 mL 1x PBS, and 1 mL FBS to a sterilized ultracentrifuge tube and centrifuging (30,000 g, 1 h, 4 °C) with low deceleration. After this spin, layers can be visually distinguished within the previously homogenous Percoll gradient solution by variations in their translucence. Using the low deceleration setting during the centrifugation, if available, supports the integrity of these layers.

After terminating the second enzyme incubation through centrifugation, the microvascular cell pellet is resuspended in 1 mL DMEM and pipetted gently against the side of the ultracentrifuge tube such that the cell solution creates a new layer on top of the existing layers and does not mix with the pre-existing layers. The ultracentrifuge tubes are then spun (1,000 g, 10 min, 4 °C) with low acceleration and low deceleration to prevent homogenous mixing and encourage layering. The Percoll gradient is useful for distinguishing more than vascular cells. Additionally, because the cell-free layers are created at the higher 30,000 g speed in the previous spin, once the cells are added the solution can be centrifuged at a lower speed which is less potentially damaging for the cells. Four distinctly colored layers are visible in the ultracentrifuge tube following the 1,000 g spin with the Percoll gradients + cells solution (Fig. 2). Both the top and bottom layers of the microvessel layer with the ultracentrifuge tube are then collected and centrifuged (800 g, 8 min). During this centrifugation step, the layers separate further, such that the endothelial and pericyte layer rises to the top of the tube and appear as a mix of red flecks within a frothy cloud layer. The red and white blood cell erythrocyte layers remain in a ring at the bottom of the tube. The frothy capillary layers are washed in culture media and plated according to the seeding ratios featured in Table 1.

#### Coatings and culture

To accomplish their in vivo roles of promoting attachment for endothelial cells, pericytes have an enhanced ability to adhere to noncoated surfaces. Additionally, their lower glucose threshold [49] allows pericytes to proliferate in nutrient media that would not support other cell types. Thus, to selectively enrich for pericytes, cells within the microvessel Percoll gradient layer are plated in uncoated non-tissue treated plates and cultured in low glucose media (DMEM (1 g/L D-glucose), 10% FBS, 50 ug/mL gentamycin sulfate). To support endothelial cell attachment, plates are coated with collagen 1 (10 ug/cm^2^), collagen IV (1 ug/cm^2^), and fibronectin (3 ug/cm^2^). Additionally, because endothelial cells interface with blood in vivo, endothelial growth media, (DMEM/F12, 10% BPDS, and 50 ug/mL gentamycin sulfate) is supplemented with plasma derived proteins, serum, and other molecules found in the blood. For this study, endothelial growth media was supplemented with heparin (0.1 mg/mL), insulin-transferrin-selenium (50 ug/mL), and bFGF (5 ug/mL). Finally, endothelial cells have unique specialized efflux pumps. Utilizing this, puromycin is added to the endothelial growth media as a selective antibiotic (5 ug/mL) which kills contaminating cells but is tolerated by endothelial cells. While it is possible to culture pericytes and endothelial cells for a longer time, it is recommended that cells used for BBB modeling be at passage 3 or below to ensure cells retain their functionality. Upon reaching confluency and phenotypic maturity, cells were fixed with 100% ice cold methanol for 10 min at -20 °C prior to imaging.

### Protocol summaries and troubleshooting resources

#### Astrocyte procedure

1. Euthanize P5 Sprague-Dawley rats via overdose with pentobarbital.
2. Spray necks with 70% EtOH, perform decapitation, and remove skull and meninges over ice.
3. Engulf brains in dissection media.
4. Remove surface vessels and place brain in DMEM with serum for mechanical dissociation via mincing with sharp scissors.
5. Triturate with 5–10 mL serological pipette (20 suspensions), then with 1 mL micropipette (10 suspensions).
6. Pass tissue solution through a 70 μm nylon cell strainer.
7. Collect strained solution, centrifuge (500 g, 5 min), and resuspend at a plating concentration of 0.2 mL per 1 cm^2^.
8. Plate on PDL or PLL coated culture vessels and culture in DMEM, 10% FBS, 1% P/S in an incubator.

#### Endothelial and pericyte procedure

1. Euthanize P63 Sprague-Dawley rats via CO_2_ exposure and overdose with pentobarbital.
2. Spray necks with 70% EtOH, perform decapitation, and remove skull and meninges over ice.
3. Engulf brains in dissection media.
4. Remove surface vessels and place brain in DMEM with serum for mechanical dissociation via mincing with sharp scissors.
5. Triturate in collagenase type II-DMEM solution (1 mg/mL) with a 5 mL serological pipette and a 1 mL micropipette.
6. Enzyme Dissociation #1: (50 min, 37 °C) incubation in a collagenase type II + DMEM solution.
7. Collect tissue solution, centrifuge (500 g, 5 min), and resuspend tissue in a 20% BSA/DMEM solution.
8. Centrifuge 20% BSA-DMEM-tissue solution (1000 g, 20 min, 4 °C).
9. Prepare 33% Percoll solution by combining 1mL 10x PBS, 9 mL Percoll ™, 1 mL 1x PBS and 1mL FBS (per brain) and centrifuge solution (30,000 g, 60 min, at 4 °C) with low deceleration.
10. Resuspend vascular pellet in a collagenase type II (0.5 mg/mL) + collagenase/dispase (2 mg/mL) + DMEM solution.
11. Enzyme Dissociation #2: (45 min, 37 °C) incubation in collagenase type II, collagenase/dispase, DMEM solution.
12. Collect tissue solution and centrifuge. Resuspend the vascular pellet in DMEM to wash.
13. Resuspend vascular pellet in 1 mL DMEM and carefully layer on top of the established Percoll gradient layers.
14. Centrifuge Percoll-tissue layers (1000 g,10 min, 4 °C) with low acceleration and deceleration.
15. Identify and collect microvascular layer. Centrifuge microvessel layer (800 g, 8 min).
16. Collect the emerged top capillary layer from 15., suspend the layer in culture media, and centrifuge (500 g, 5 min).
17. Resuspend the pellet resulting from Step 16. in desired plating concentration.
*Pericytes: Plate on uncoated plate with low glucose DMEM.
*Endothelial Cells: Plate on collagen 1, collagen IV, and fibronectin coated plate with high-glucose DMEM-F12 with BPDS, supplemented with heparin, insulin-transferrin-selenite, basic fibroblast growth factor, and selective antibiotic puromycin.

#### Troubleshooting resources

Additional procedural recommendations to further increase the yield of neurovascular cells include keeping reagents and cell pellets between processing steps on ice as much as possible to preserve viability throughout the lengthy vascular isolation processes, with the exception of enzymatic digestion steps. Scissors, tubes, and vials can be coated with serum or proteins to decrease cell attachment to tools and increase overall yield [31]. During trituration steps, it is important to minimize excessive force and speed of suspensions which can result in undo shear on cells and lead to lysis of cell membranes. Reducing the force and speed of suspensions also reduces bubble formation which can prevent tissue homogenization and exposure to digestion enzymes. Over-trituration can be avoided by pipetting around 20 times up and down first with a serological pipette then around 10 times with a micropipette [4, 38]. In our experience, it is beneficial to process at least two brains in parallel so that the tissue can rest from isolation trauma while the other brain is processed. For example, in the present work, the tissue-enzyme-media solution from one brain was triturated first with 5 up and down suspensions. Then, the tissue- enzyme-media solution from the other brain was triturated with 5 up and down suspensions while the first brain tissue rested in the enzyme solution. The processing then alternated between brains until approximately 20 suspensions had been achieved for each brain. An additional benefit to processing two brains together is that the resulting final pellets are larger and more visible and thus easier to identify. It is possible to use younger pups (P21-P35) to obtain endothelial cells and pericytes, but our overall yield was greater from the larger P56-P63 brains, though upper age limits beyond this were not investigated in our study.

The type and concentration of enzymes used in the presented protocols were optimized to avoid over-digestion while obtaining pure cultures of the cells of interest. When DNAse was used in our pericyte and endothelial isolation procedures, the final cell solution was observed to have a higher percentage of cell fragments and debris compared to cells with a round appearance which indicated that the culture had a low percentage of cells with whole and intact membranes. These initial results informed our decision to reduce the enzyme concentration. When collagenase type II was used alone, the debris seen in the pericyte culture after attachment at 2 DIV was greater indicating that more digestion was needed to separate and remove non-pericyte cells that ended up in the final solution and died due to insufficient nutrients. The final resulting enzyme combination of collagenase type II and collagenase/dispase was chosen based on their biological targets for digestion, the clarity of the resulting post isolation cell suspension, and the clarity of the first few days of pericyte attachment. This optimization was then applied to the isolation of endothelial cells and led to more microvessels remaining in the final cell solution, more endothelial cell patches observed at 1 DIV, and confluency being reached more rapidly. The 20% BSA separation step was also an early indicator to determine if the extent of homogenization and digestion steps were too extreme. In trials where no thin layer of undigested tissue was seen at the top of the conical, the overall cell yield at the end of the isolation was lower. In trials where a thin line of undigested tissue was seen, the final number of cells plated was higher and cells proceeded to proliferate. However, large quantities of undigested tissue may retain microvessels, entrapping and preventing them from being separated into the final pellet, resulting in a lower number of overall microvessels. Thus, a thin layer of undigested tissue is optimal.

Reducing non-attaching cell death and debris in culture vessels can increase overall cell yield and viability by reducing stress in the plate environment for target cells. Additional cell separation steps such as the 70-µm filter in the astrocyte isolation and separating out erythrocytes from endothelial cells and pericytes is one way to accomplish this. These separation steps also allow greater attachment as cells of interest have less obstacles in the form of non-attaching cells to navigate as they settle at the bottom of the plate. Though the initial astrocyte media change can be done at either 2 DIV or 3 DIV, it is recommended to change media as soon as cells of interest are fully attached out of the original cell suspension to reduce the stress from debris in the culture environment. If cell attachment is slow, it is possible to collect and spin down previous media to create a pellet which can be resuspended and added in part into the vessel with fresh media containing unspent nutrients. Media collected and spun down at 2 DIV for endothelial cells, results in a pellet that can be washed, to remove the puromycin, and plated on uncoated plates to produce viable pericytes, though overall yield will be lower compared to dedicated pericyte isolations.

Iterations on neurovascular cell isolation protocols over the past 20 have resulted in advancement such as reducing pericyte contamination of endothelial cells and microglia contamination of astrocytes and increasing overall cell viability and purity. However, these iterations have also led to the field becoming saturated with a variety of practices without guidance on whether these alternatives are helpful or outdated and thus less effective. Though the methods presented here have reproducibly and reliably been used to isolate neurovascular cells in numerous trials, consideration of the variations used by other groups may be warranted depending on the purpose of the isolated cells and to address inherent lab to lab variability and equipment or reagent limitations. Common variations to the procedures used in the present study are captured in Table 2. Demonstrated by the number of references by each step, most protocols agree on the necessity of the initial meninges removal, mincing, and trituration steps, the use of two rounds of enzymes, the 20% BSA separation and the Percoll separation steps. Thus, in the presented work, these steps are highlighted along with our rationale. Most protocols differ distinctly in the type of enzyme used, duration of incubation, and concentration used. If cells become lysed or damaged during mechanical dissociation, fragments of DNA can become exposed and cause cells to stick to one another. For this reason, many protocols include DNAase 1 to degrade these DNA fragments. However, with the careful processing steps featured in our protocol, such as measured mincing and trituration and reduced digestion times, we have found this enzyme to not be a requirement. To prevent contamination of meningeal cells, many groups have included the use of filter paper or other sterile mesh-like materials onto which the brain tissue is rolled leaving the meninges adhered to the material. For further purity of cultures, several groups employ sequential plating based on the different time periods required for different cell types to migrate and settle at the bottom of a culture vessel. Using sequential plating, a mixed cell solution may be allowed to adhere for a short duration while cells that remain floating in the suspension are collected and plated separately. This strategy has been used to separate adherent microglia and oligodendrocytes from suspended astrocytes and pericytes as well as to separate adherent microglia from suspended oligodendrocytes [13, 37, 39]. To enhance endothelial barrier properties, several groups have cultured neurovascular cells in media containing hydrocortisone to promote tight junction formation [4, 37, 42]. For additional methods used in other protocols that may assist in troubleshooting neurovascular cell isolation outcomes, it is recommended that the process steps featured in Table 2 be used as a starting point for elements to incorporate in isolation processes.

## Isolated neurovascular cell growth in culture over time

To characterize primary neurovascular cell growth after isolation and plating, each cell type was monitored over 12 DIV; cells were evaluated via phase contrast imaging each day in culture and astrocyte glial cultures were fixed and imaged at 3 DIV, 5 DIV, 10 DIV, and 12 DIV. The day of isolation is referred to as 0 DIV.

### Astrocyte glial culture

**Fig. 3:**
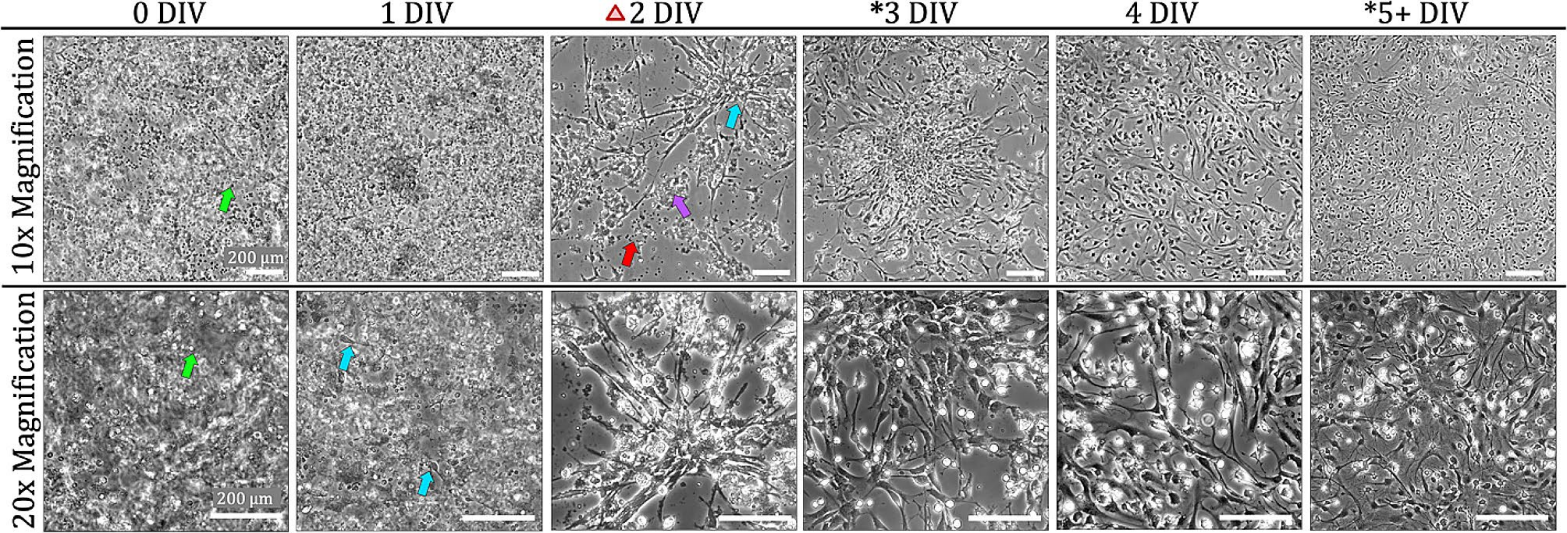
Astrocyte growth in culture: Single cell suspensions are visible (green arrow) even without using enzymatic digestion in the isolation process. At 1 DIV, small cell somas can be seen attached to the bottom of the plate with 1–2 branches beginning to form. By 2 DIV, the first media change is completed as indicated by the red triangle icon. Cell debris from dying non-astrocyte cells are depicted by red arrows. Other non-attached cells can be seen (purple arrow). The blue arrow shows many cell bodies growing in the same area forming snowflake-like clusters. These clusters can be seen extending through culture days. At 5 DIV, cells are passaged, and morphology remains heterogeneous, but does not change with more time in culture. Asterisks indicate timepoints where confocal imaging was completed. Scale bars in all images: 200 μm

Though most of the brain tissue is plated after the initial astrocyte isolation, single, rounded cell bodies can be seen settling at the bottom of the culture vessel (Fig. 3). Astrocyte attachment can be seen at 1 DIV appearing as small, flat, opaque, and adherent structures that do not move when the rest of the flask contents are jostled or rocked. These structures often appear with one thin projection that then becomes the first branch of the newly attached cell. The non-attached contents of the flask are the remains of non-glial cells and connective tissue. The first media change is conducted at 2 DIV where much of the non-attached flask contents are rinsed away. At this point, cell attachment is quite apparent with branches extending from cell bodies gathered in clusters throughout the culture vessel. After the media change, some of non-attached contents remain entangled with the attached cells at 2 DIV. Non-attached cells and debris are still visible at 3 DIV but there are distinct cells underneath growing in snowflake-like clusters. Cells begin to grow between clusters to connect with each other with increased culture time. The first passage is conducted at 5 DIV based on the 80% confluency reached at this stage. In contrast to the cell behavior after original isolation, astrocyte attachment to culture vessels after passaging is evenly distributed throughout the plate without the formation of the distinct cluster shapes.

### Pericyte culture

**Fig. 4:**
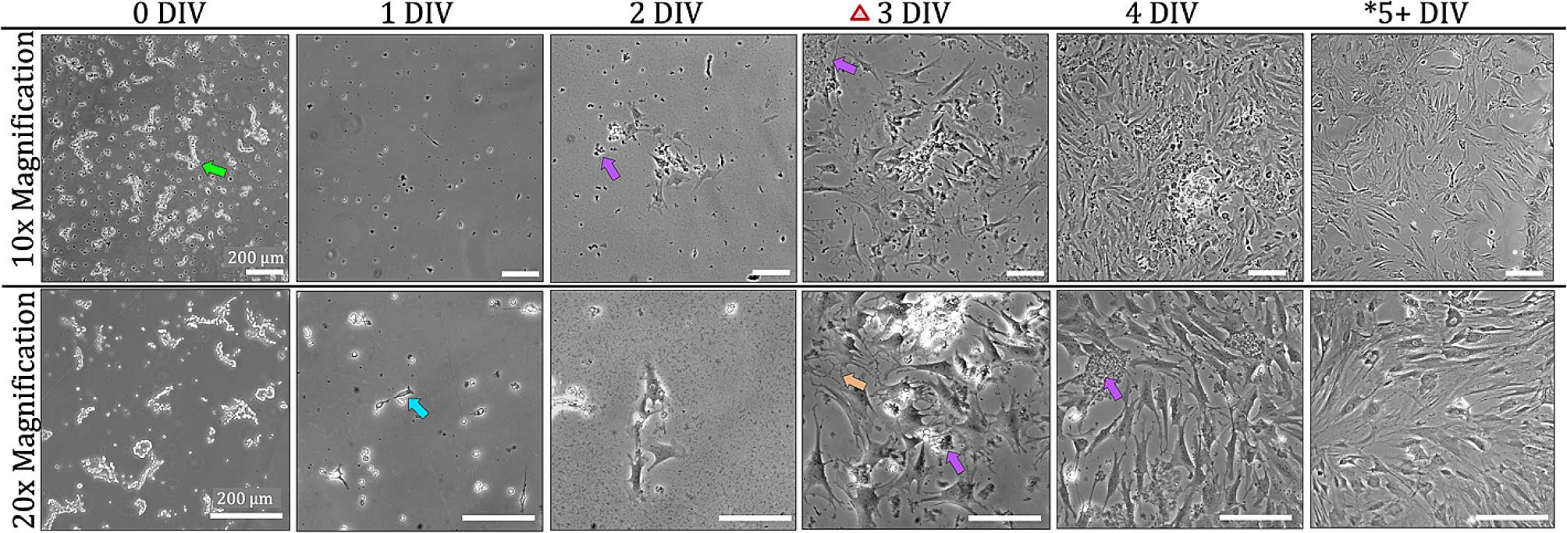
Pericyte growth in culture. Immediately after isolation cell clusters are visible (green arrow), where individual cells are distinctly visible, but microtubules remain intact. At 1 DIV, a few small cell somas can be seen attached to the bottom of the plate. By 2 DIV, the first media change is completed as indicated by the red triangle icon. Pericyte-specific morphology is seen as early as 2 DIV characterized by polygonal cells with finger projections. Cell debris (purple arrow) from dying non-pericyte and other non-attached cells are visible in areas of the plate where cells are not attached whereas the spaces between attached cells are clear. Extensions of pericyte processes (orange arrow) are visible as pericytes attempt to form connections among other pericytes. At 7 DIV, cells are passaged, and morphology does not significantly change with more time in culture, cells become larger, flatter, and proliferate. Asterisks indicate timepoints where confocal imaging was completed. Scale bars in all images: 200 μm

The resulting cell solution following a pericyte or endothelial isolation contains groups of cells clustered together that appear as pearls on a string (Fig. 4). These groups of linked cell bodies are endothelial cells within intact microstructures. In addition to the clusters, single cells can be seen and will contribute to the eventual pericyte population. Early pericyte attachment is visible at 1 DIV and appears as described in the Astrocyte Glial Culture section. However, early pericyte morphology begins to show distinct differences from early astrocytes as early as 2 DIV. Whereas astrocytes have smaller somas with long, thick, branches, pericyte somas comprise the majority of the cell area, with small fine branches extending from the edges. The remaining non-pericytes cells within the mixed microvascular cell solution that cannot attach to an uncoated surface or survive in the low-glucose culture environment will eventually die and lead to an accumulation of cell debris in the culture vessel that becomes visible at 2 DIV. This debris covers all areas of the plate where cells are absent, but areas of the plate with adherent cells are clear of debris. At 3 DIV the debris becomes loose and gathers in clumps. After the media change is performed on 3 DIV, the cells attached to the bottom of the vessel exhibit pericyte-specific morphology with rhomboid shapes and thin finger-like projections. Pericytes can be seen growing in non-overlapping patches that connect and communicate through long thin branches extending from cell bodies (Fig. 4).

As the number of cells increase due to pericyte proliferation, the debris clears completely around 5 DIV. While early pericyte cultures are spindled with thin branches on either side of the nuclei, as pericytes mature, they eventually flatten with increased culture time becoming more transparent with somas widening in circular, sheet, standard, and rhomboid shapes in agreement with previously established pericyte morphology characterization [23]. Other characteristic pericyte phenotype includes jagged edges, as opposed to rounded cell bodies, and projections that extend at angles. Pericyte morphology is influenced by the confluency of the culture vessel, with pericytes in lower confluency settings expanding in width and extending thin processes to cover more area and connect with other cells. Pericytes become thinner and more elongated in response to higher confluency.

### Endothelial culture

**Fig. 5:**
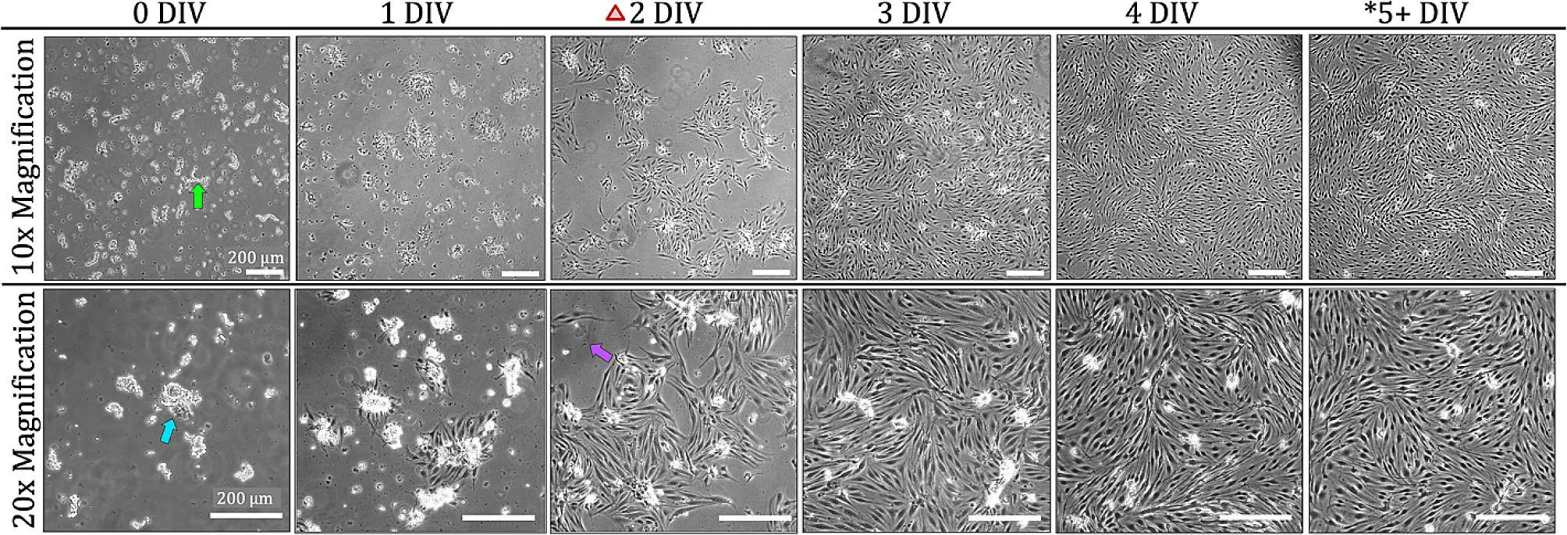
Endothelial growth in culture. Immediately after isolation, cell clusters are visible (green arrow), where individual cells are distinctly visible, but microtubules remain intact. 2 h after isolation 0 DIV, cell somas (blue arrow) can be seen attached to the bottom of the plate around the cell clusters. By 2 DIV, the first media change is completed, indicated by the red triangle icon. Endothelial-specific morphology is seen as early as 0 DIV characterized by cells growing in circular patterns extremely close to one another. At 1 DIV, cell debris from dying non-endothelial and other non-attached cells are visible (purple arrow). Each endothelial cluster grows and extends to combine with other clusters. Depending on plating concentration, cells reach confluency and can be frozen as early as 5 DIV. Morphology does not change between 0 DIV and 1 DIV, cells simply become longer with the same phenotype with increased culture time. Scale bars in all images: 200 μm

As the isolation process is conserved between endothelial and pericyte isolations, the appearance of the initial cell solution contains the pearls on a string described in Pericyte Culture section. However, the cumulative effects of the supplemented media and plate coatings on cell attachment can be seen within 3 h after isolation on 0 DIV. At 3 h post-isolation, small, flat, pancake-like structures can be seen at the edges of some cell clusters (Fig. 5). Adhered cells are differentiated by their wider shape, fringe edges, and color – as the adhered cells feature a dark nucleus, transparent body, and dark surrounding cell membrane while unattached clusters appear bright white when viewed with a phase contrast microscope. The early adhered cells already display endothelial morphology of a centralized nucleus within a rounded cell body closely attached to other cells in a circular cluster. At 1 DIV, multiple small patches of cells packed tightly in spirals are seen evenly distributed throughout the vessel. Most of the cell clusters have a degree of endothelial attachment at the edges with cells at the center of the clusters not yet attached while some clusters of endothelial cells become fully adherent and elongated. After 0 DIV, few cells retain the round pancake shape – the majority of cells appear as long fusiform ellipses.

In culture, endothelial cells simply become longer and thinner with increased contact with one another. The endothelial cell clusters grow in spirals and when the clusters become large enough, between 40 and 60 cells in each cluster, adjacent clusters combine, and some cells change direction of their growth to match the spiral direction of the adjacent cluster. Due to the selective antibiotic, non-endothelial cells die and become debris which is then cleared from the plate through media changes. Endothelial growth after isolation is rapid and depending on initial plating concentration, endothelial cells can become confluent in as few as 5 DIV. Pericyte and endothelial cell morphology and growth timelines differ significantly despite sharing the initial isolation procedures.

**Fig. 6:**
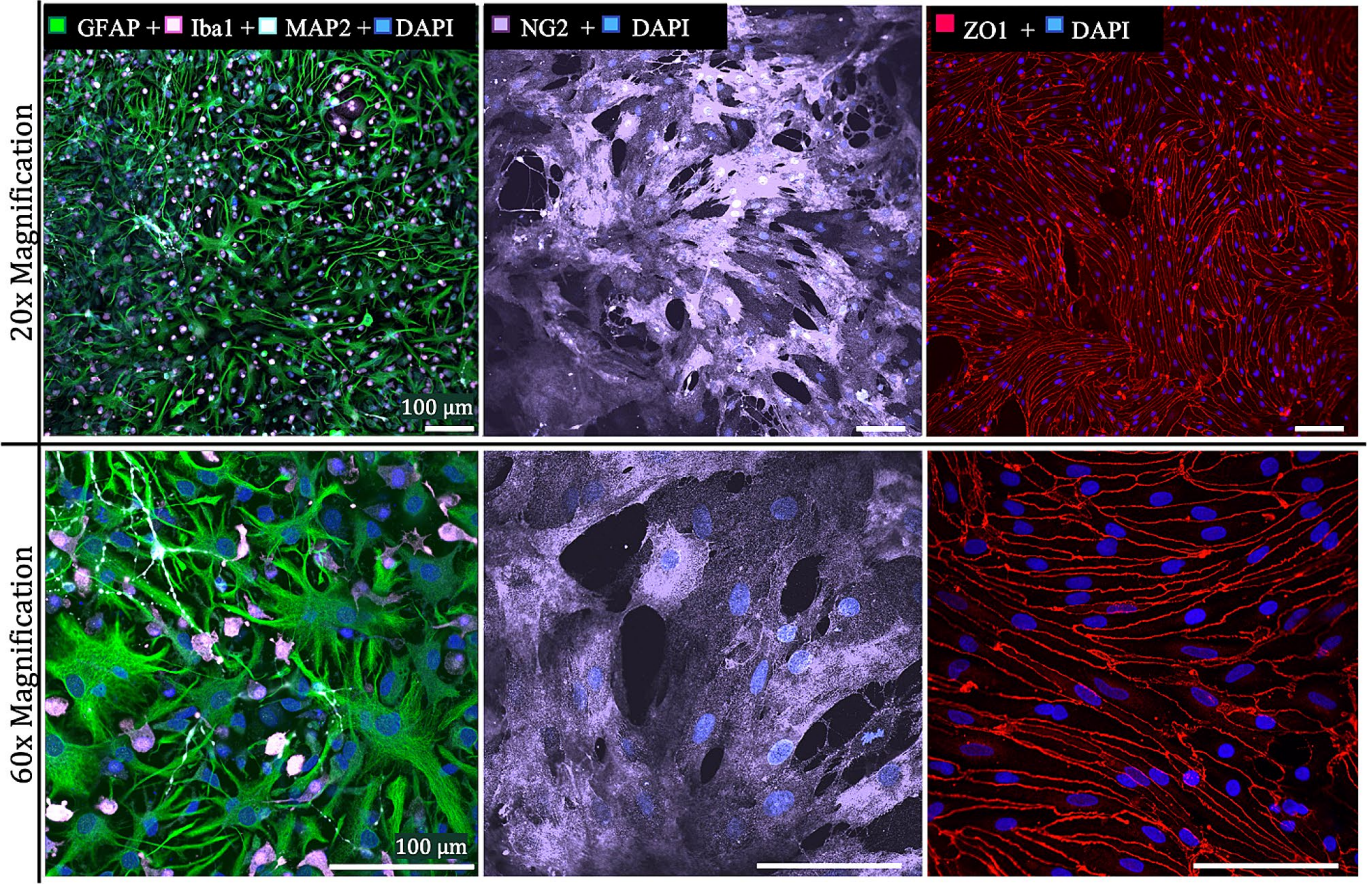
Immunocytostaining for cell identity. *Left*: *GFAP* + astrocytes (green), *Iba*1 + microglia (pink), and *MAP2* + neurons (cyan) are presented in culture at 12 DIV. *Middle*: *NG2* + pericytes (purple) show rhomboid phenotype with cells growing on top of one another in multiple Z planes at 12 DIV. *Right*: ZO-1 + endothelial tight junctions (red) form between endothelial cells at 5 DIV. All visible nuclei are *DAPI*+ (blue). Scale bars in all images: 100 μm

Upon reaching confluency, cells are immediately used in experiments or frozen down for future use. By the time confluency is achieved for each cell type, mature morphology is confirmed through visual analysis of astrocyte branching complexity, rhomboid structure and fine processes of pericytes, and fusiform shape, length and directional growth of endothelial cells (Fig. 6). Confirmation of each cell type was done using GFAP + cells to indicate astrocytes, NG2 + cells to indicate pericytes, and ZO-1 + cells to indicate functioning, tight junction producing endothelial cells as validated in previous literature.

## Glial culture composition over time

**Fig. 7:**
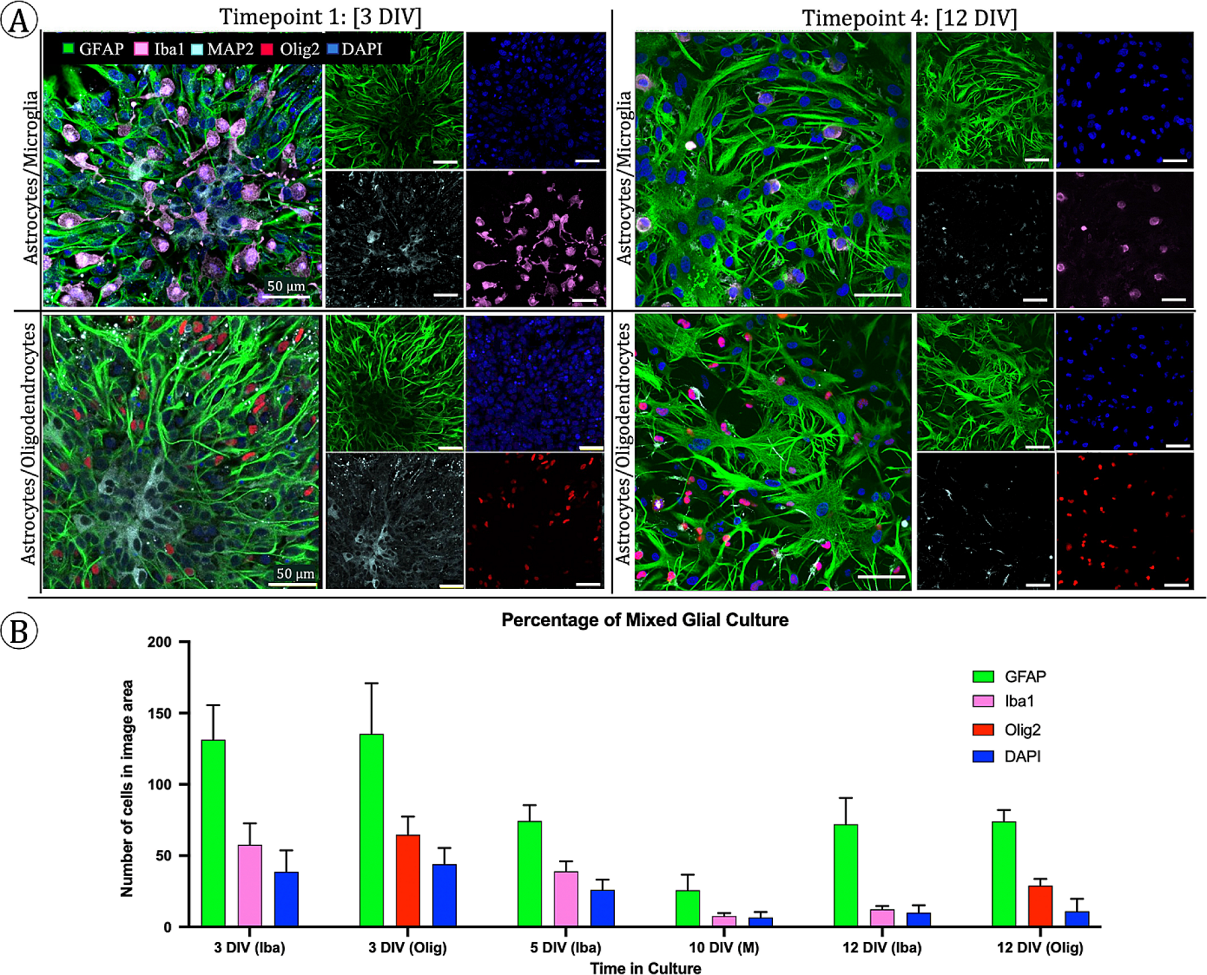
Presence of microglia, neurons, and oligodendrocytes. **(A)** Top: GFAP + astrocytes (green), Iba1 + microglia (pink), and MAP2 + neurons (cyan) are presented in culture at 3 DIV and 12 DIV. Microglia are visible in both time points but decrease in percentage and change morphology to become less branched at 12 DIV. Bottom: GFAP + astrocytes (green), Olig2 + oligodendrocytes (red), and MAP2 + neurons (cyan) are present in culture at 3 DIV and 12 DIV. The relative number of Olig2 + cells does not appear to change between timepoints. In both images at 3 DIV, some MAP2 + neurons are visible with the number and size of neurons decreasing by 12 DIV. All visible nuclei are DAPI+ (blue). Scale bars in all images: 50 μm. **(B)** Glial populations are mostly astrocytes at each time point with the fraction of microglia to total cells decreasing at 10 DIV and 12 DIV and the fraction of oligodendrocytes to astrocytes remaining relatively constant. The DAPI refers to all nuclei that is neither one of the two other cell types analyzed in each group

Mixed astrocyte glial cultures were fixed and co-stained for GFAP, Olig2, and MAP2 at two timepoints, 3 DIV and 12 DIV, corresponding to early and late within their growth process to further investigate the presence of neurons and oligodendrocytes within the culture. Another set of mixed astrocyte glial cultures were fixed and co-stained for GFAP, Iba1, and MAP2 at four timepoints, 3 DIV, 5 DIV, 10 DIV, and 12 DIV to further investigate the presence of neurons and microglia within the culture. The four timepoints corresponded to distinct shifts in morphology as assessed via daily phase contrast imaging. Microglia, neurons, and oligodendrocytes were present in and constituted a considerable portion of the culture at 3 DIV, yet these cells were outnumbered by the number of astrocytes (Fig. 7). At 12 DIV, microglia were still present in the culture, but at a lower ratio and with less branching. This was expected as microglia were not being enriched for during culturing. Oligodendrocytes were also still present in the mixed culture at 12 DIV at a similar ratio as they were at 3 DIV. Few neurons were seen at 12 DIV compared to the 3 DIV timepoint and those that did remain did not exhibit the morphology seen in 3 DIV and appear smaller and potentially fragmented which is expected as culture conditions were not specific to support neurons. Areas of similar confluency were analyzed at each timepoint. However, because the distribution of adhered cells on the surface of the culture vessel after passaging was even across the plate instead of clusters, the number of cells that can be seen in a given imaging area can appear lower after passaging. Additionally, cells after passaging have larger somas and branches thus the same confluency of cells is reached with fewer overall number cells at 10 DIV and 12 DIV compared to 3 DIV and 5 DIV. Thus, our analysis is expressed as a percentage of the total cell population in the given imaging area as opposed to total number of cells.

At 3 DIV, the percentages of astrocytes in cultures are between 55 and 58%. At 5 DIV, the percentage of GFAP + cells remains similar (53%) and increased to 64% at 10 DIV. For cultures co-stained with Iba1, GFAP + cells were 76% of the identified cells at 12 DIV. For culture co-stained with Olig1, GFAP + cells were 60% of the total identified cells reflecting the higher percentage of Olig2 + cells compared to the percentage of Iba1 + cells. The ratio of GFAP + cells to Iba1 + cells adjusts slightly in favor of the GFAP + cells while the ratio of GFAP + cells to Olig2 + cells remains more constant.

**Fig. 8:**
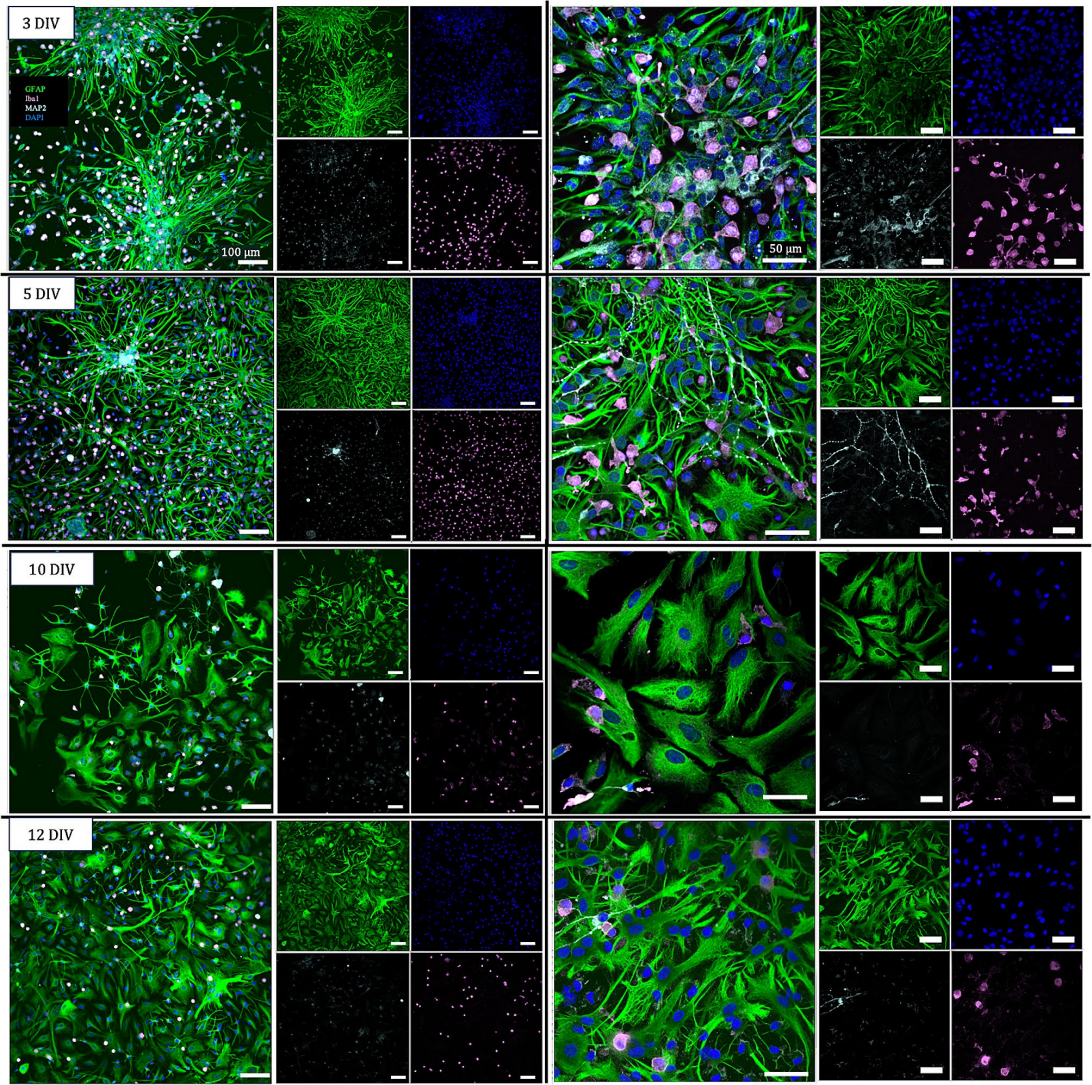
Glial distribution and morphology over time. GFAP + astrocytes (green), Iba1 + microglia (pink), and MAP2 + neurons (cyan) are present in culture at 3 DIV, 5 DIV, 10 DIV, and 12 DIV. At 3 DIV, large clusters of individual astrocytes are seen. Astrocytes are relatively simplistic in shape mostly with a branch extending on each end on the nucleus. Microglia at 3 DIV have different degrees of branching. At 5 DIV, cells begin to have > 2 branches while large clusters of individual astrocytes remain. Simplistic astrocytes are seen along with more mature astrocytes with thicker, more fibrous cell bodies with multiple branches. Microglia at both 3 DIV and 5 DIV present with various degrees of branching. At 10 DIV, classical astrocyte morphology is seen, and cells are evenly distributed over the growth area instead of in clusters. Astrocytes appear thick with projections of various sizes while microglia are less numerous and rounder. At 12 DIV, cells begin to grow to confluency, yet the number of cells is lower as each cell is larger and takes up more space than the cells in 3 DIV and 5 DIV. Astrocytes have a mix of thick and fibrous and thin stars-like shapes but none of the earlier more simplistic shapes. Microglia only appear in round shapes. All visible nuclei are DAPI+ (blue). Scale bars in all images in left column: 1000 μm. Scale bars in all images in right column: 50 μm

We show that astrocytes produce a GFAP + response as early as 3 DIV despite lacking the complex branching morphology consistent with mature astrocytes (Fig. 8). DAPI staining implies that the early astrocyte clusters are comprised of many astrocytes with one to two branches. Although adhering within clusters initially, cells appear to attempt to bridge the clusters together. Additionally, neurons that were present in the culture appeared to localize in the center of the astrocyte clusters in areas of lower relative GFAP + expression (Fig. 8). Microglia were evenly distributed among the astrocytes and oligodendrocytes appeared to remain further from the center of the astrocyte clusters. At 5 DIV, the cluster shape can still be seen along with astrocytes extending branches to connect with cells beyond the clusters as well as cells growing between the gaps.

Cells were passaged at 5 DIV and adhere with even distribution across the growth surface as seen in the 10 DIV and 12 DIV timepoints. While 3 DIV and 5 DIV are dominated by one to two branched astrocytes, astrocytes with the characteristic fibrous and protoplasmic phenotypes begin to emerge at 5 DIV. These phenotypes then become the dominant morphology at 10 DIV, while more complex branched astrocyte morphology appears at 12 DIV. With increased branching and wider cell bodies, each cell inhabits more space on the growth surface and cells do not overlap to the extent that they appear to at 3 DIV. Compared to the clusters at 3 DIV, each star-shaped structure at 12 DIV is a single cell with many branches as opposed to many cells with single branches. Microglia retain their branches at the 3 DIV and 5 DIV timepoints but appear to shift to a more ameboid morphology after the first passage and subsequent culture.

Overall, these results show our ability to obtain pericytes, endothelial cells, and mixed glial cultures consisting predominantly of astrocytes that include distinct supporting neurovascular unit cells as confirmed via immunostaining. The breadth of morphologies throughout culturing time over multiple trials are captured in Supplemental Figs. 4–7. Image analysis of GFAP+, Iba1+, and Olig2 + cells revealed that the composition of the mixed glial culture includes a larger percentage of astrocytes than microglia and oligodendrocytes at each timepoint in culture with the percentage of microglia decreasing at the timepoints after passage.

## Discussion

The presented protocols resulted in a high yield of astrocyte dominant glial cells, pericytes, and endothelial cells with high purity and established morphological metrics at key stages of growth. Through reduced enzyme usage, gentle processing steps, and effective separation techniques, we reduce the amount of non-attaching cell death and debris in culture vessels which allows for increased viability of our target cells. The small number of debris that was not removed in our process steps appears to lessen with culture time in astrocyte and pericyte cultures indicating that these cells are expressing some level of clearance mechanisms.

There remain limitations in the present study and with primary cell isolation more broadly. Primary cells begin to lose functionality after multiple passages which limits the total amount of cells that can be obtained from one isolation for use in in vitro BBB modeling. While astrocytes should not undergo more than three passages, they can be split at high ratios during the first two passages and thus produce the highest overall yield compared to pericytes and endothelial cells which are limited in both number of passages and proliferation. In this study, astrocytes were cultured until passage 2. Cell morphology and identity of cells after passage 3 may continue to change but were not discretely investigated in the present study. The lack of neurons and microglia present in our mixed glial cultures at later timepoints and the change in microglia to non-branching morphology could be due to lack of enrichment in the culture media components for these cell types. Though endothelial cells and pericytes can be isolated from the same brain using sequential plating techniques, it remains difficult to isolate astrocyte/glial cultures from the same brain as the proliferative capacities of these astrocytes are more pronounced at the neonatal age whereas endothelial cells and pericytes retain their in vivo morphology and function better when isolated from more developmentally mature brains. Though it is outside the scope of the current work, it is also worth acknowledging the shortcomings of culturing cells in 2-dimensional plastic growth surfaces. Although a common culturing environment, the lack of physiologically relevant architecture inherently applies stress to cells and can increase cell reactivity [14].

GFAP is a validated and commonly used marker to identify astrocyte populations, however, it does not capture all astrocyte morphology. Additional information could be captured by staining for astrocyte markers such as S100B, Aquaporin-4, GLAST-1, and other glial cell markers. Similarly, Olig2 only stains cell somas so we cannot make determinations about branches and changes to morphology throughout culture time or differentiate between oligodendrocyte precursor cells and mature oligodendrocytes. Growing evidence has also shown that staining pericytes for NG2 along with PDGFR-β and aSMA allows for greater specificity and insights into pericyte lineage to be ascertained [23, 39]. An additional shortcoming of the present study is that only positive cell staining was used as opposed to including negative staining to further prove cell identity and purity of cultures. Future work could integrate single cell sequencing to further study cell-cell interactions that are beyond the focus of this set of protocols.

With the analysis techniques employed, we establish baseline metrics of morphology and phenomic standards for various neurovascular cells in 2D culture at different time points post isolation. These protocols include a resource of morphological benchmarks to evaluate the effectiveness of primary neurovascular cell isolation alongside a compiled repository of troubleshooting steps and alternatives for labs that may or may not have access to transcriptomics or sequencing. Clearly defined protocols with the ability to reproducibly obtain these cell types with expected morphology will help provide source material to train data science or other quantitative image analysis platforms to standardize the emerging area of phenotypic analysis. Phenomic analysis can be integrated with metabolomics, proteomics, trancriptomics, and epigenomics data to get the most complete picture of the in vitro functionality of neurovascular cells.

Accurate in vitro disease modeling requires the inclusion of multiple cell types so the contributions of cross talk in pathogenesis and disease progression can be captured. Reactive astrogliosis, though named for the role of astrocytes, is worsened through cross talk and interactions between microglia, oligodendrocytes, neurons and astrocytes featured in ischemia, Parkinson’s disease, and other states of brain injury [19]. There is increased data implicating the role of astrocytes in the progression of Alzheimer’s disease and multiple sclerosis (MS) through interactions with microglia and role in the neuroinflammatory response [9, 17]. Having the ability to isolate these cell types together along with the ability to visualize them distinctly enhances the use of in vitro models for investigation of cell interactions. Additionally, with multiple cells contributing to disease, in vitro models can be used to evaluate cell-specific therapeutic association and effect, and subsequent changes in the brain microenvironment, which can inform drug targeting, development, and delivery strategies.

The specific roles that pericytes play in BBB maintenance and modulation as well as the neuro-immune axis is still relatively undefined - underscoring the outstanding need for reproducible and high-yield methods of isolating this cell type. However, increasing evidence indicates pericytes as important responders to ischemic events, releasing chemical signals that are amplified by microglia and lead to astrogliosis [11, 16]. Without microglia in in vitro models of the BBB, serving as important mediators for disease progression, potential discrepancies between in vitro models and in vivo conditions can be magnified. By accurately quantifying and documenting the microglial content, as demonstrated in our work, researchers can incorporate microglia into their mixed cultures with greater confidence and enhance the fidelity of disease models.

## Conclusion

We develop protocols that successfully isolate neurovascular cells with high yield. We obtain a mixed culture of glial cells predominately comprised of astrocytes, a pericyte culture, and endothelial culture, and demonstrate features of their attachment, growth timelines, and characteristic morphology through the first week of culture. Finally, we identify the presence of neurons, microglia, and oligodendrocytes in the mixed glial culture and document how these cells change in both number and morphology over 12 days of culture. It can be beneficial to isolate mixed glial cultures in addition to astrocytes for use in in vitro drug delivery screening to assess selective uptake by specific types of cells. Astrocytes, microglia, neurons, and oligodendrocytes engage in frequent and complex cross talk in vivo and can influence cell characteristics in response to inflammation or other pathological conditions. Thus, to ascertain more clinically relevant results on in vitro drug screening, in vitro assays should include astrocytes in communication with other glial cells. In addition to use in in vitro drug delivery studies and investigations of pathogenesis, cells obtained through these protocols can be used to produce conditioned media for the collection and analysis of extracellular vesicles and to condition other cell types such as neurons or endothelial cells. Overall, we expect the primary isolation protocols to be implemented by researchers who aim to incorporate primary neurovascular cells into in vitro modeling pipelines. This work enables primary cells to be used more reproducibly and widely in BBB models to investigate the effects of disease, environment, and genetic history on therapeutic transport across the BBB.

### Electronic supplementary material

Below is the link to the electronic supplementary material.

Supplementary Material 1

## Data Availability

No datasets were generated or analysed during the current study.
