# Supplementary material for: Isolation methods and characterization of primary rat neurovascular cells
Source: J Biol Eng. 2024 Jul 11;18:39. doi: 10.1186/s13036-024-00434-3 (PMC11241874; doi:10.1186/s13036-024-00434-3)
Supplement: Supplementary file 1 — Supplementary Material 1 [file 13036_2024_434_MOESM1_ESM.docx]

**SUPPLEMENTARY INFORMATION**

**
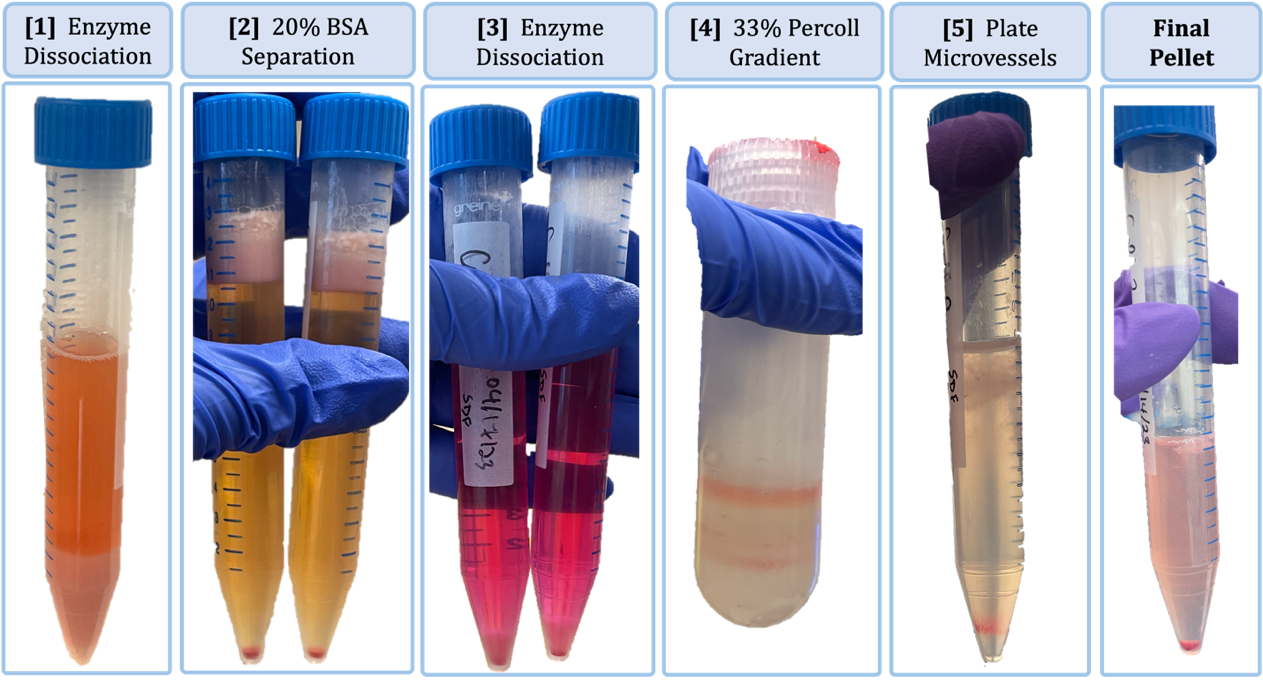
**

**Supplementary Figure 1: Real-Time Images of Critical Microvascular Separation Steps:** Each step corresponds to the artistic rendering featured in Figure 2. A blue or purple glove can be seen holding up many of the tubes. Steps 1 – 3 and Steps 5 and 6 all depict 15 mL Falcon conical tubes. Step 4 depicts a 30 mL ultracentrifuge tube.


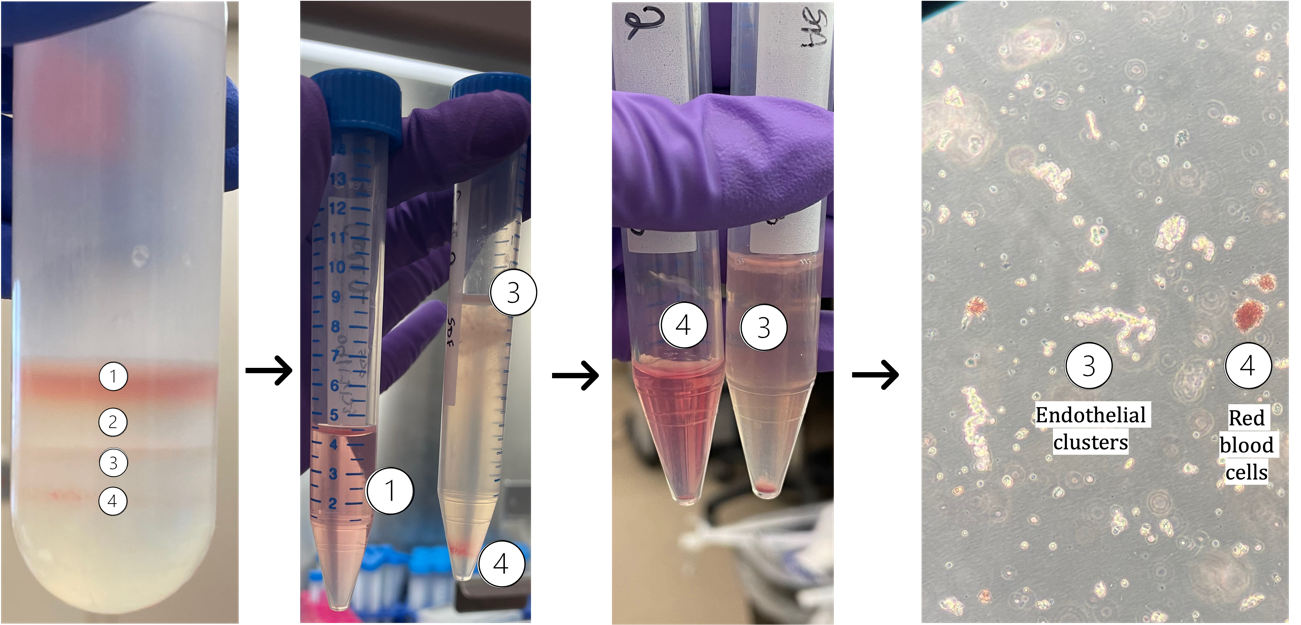


**Supplementary Figure 2: Identification of Percoll Layers:** Each layer following the Percoll gradient were collected and spun down until final pellets were obtained. When the pellet from layer 3 was resuspended, plated, and checked under an inverted microscope, endothelial clusters were seen. When the pellet from layer 4 was resuspended, added to the same plate, and re-checked under an inverted microscope, clusters of red blood cells were now seen. This work influenced the distinction featured in Step [5] of Figure 2.


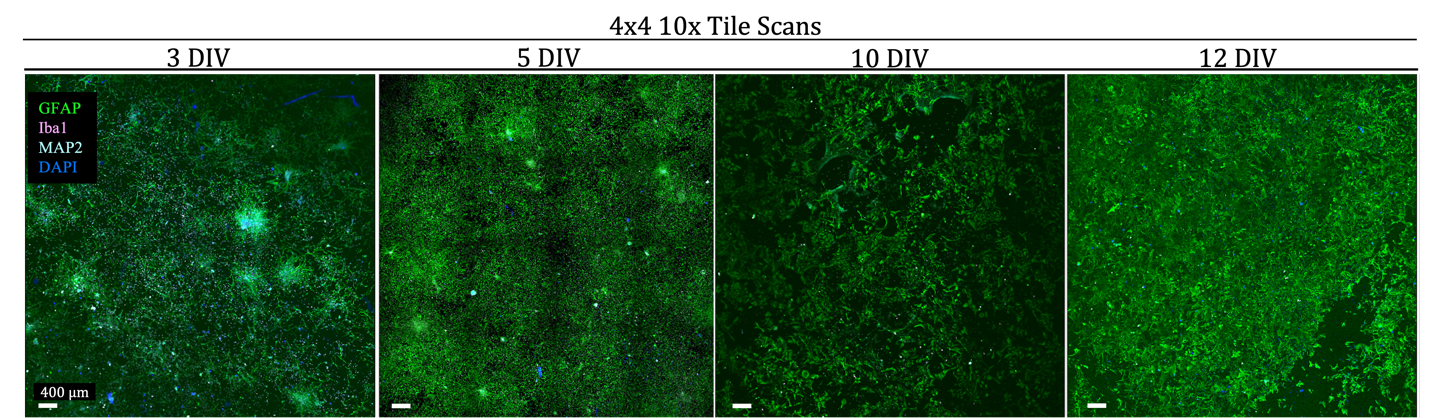


**Supplementary Figure 3: Cell Distribution at Each Timepoint:** These images were 4x4 tile scans taken at 10x on a Nikon Confocal. Each image of 16 stitched 10x tiles shows how cells are distributed across the growth area of the culture dish and confluency. Scale bars in all images: 400 μm.


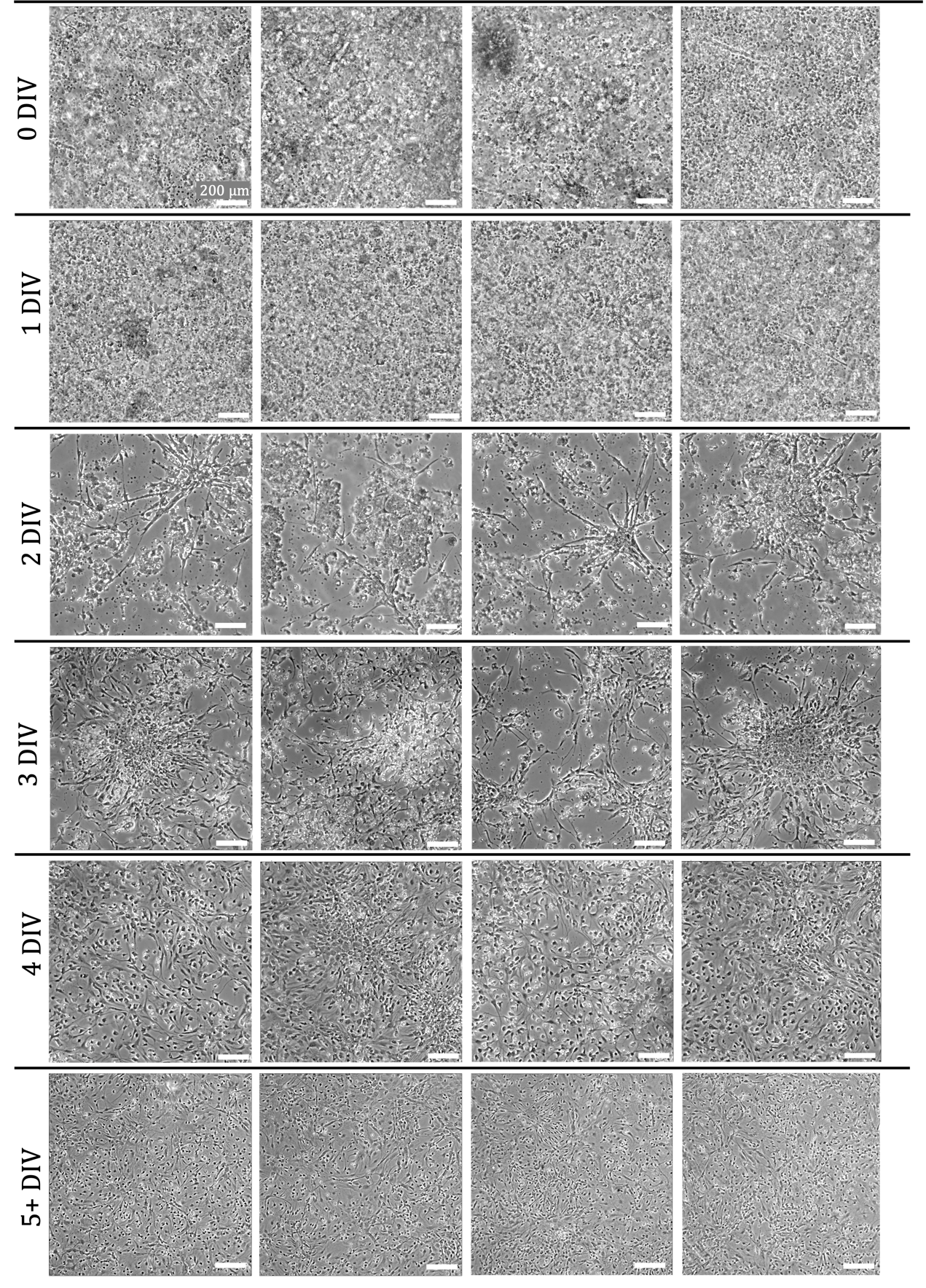


**Supplementary Figure 4: Mixed Glial Culture Growth:** These images document the range of morphology exhibited by primary isolated mixed glial cultures. Each image was taken with phase contrast microscopy and scale bars are 200 μm.


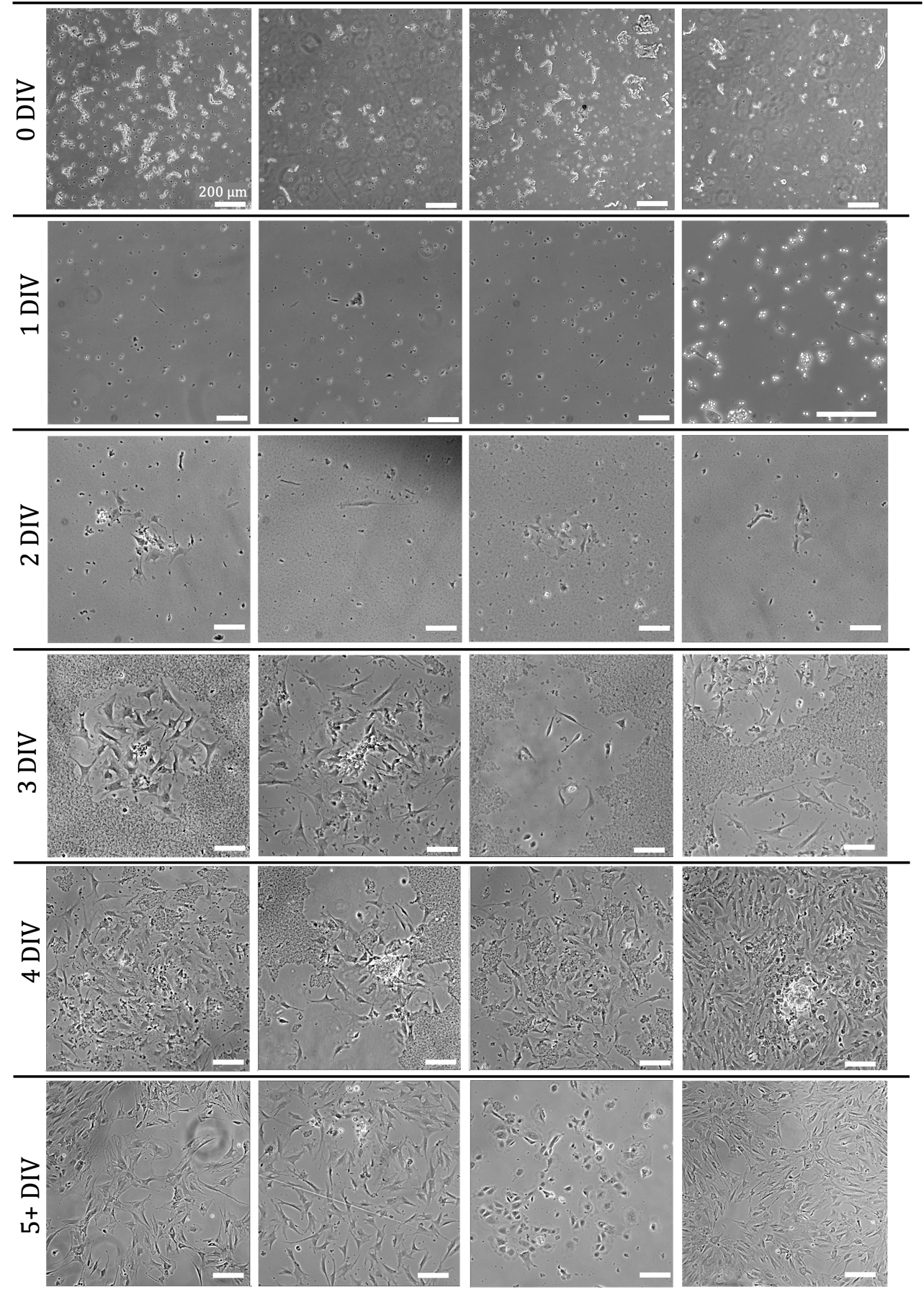


**Supplementary Figure 5: Pericyte Culture Growth:** These images document the range of morphology exhibited by primary isolated pericyte cultures. Each image was taken with phase contrast microscopy and scale bars are 200 μm.

**
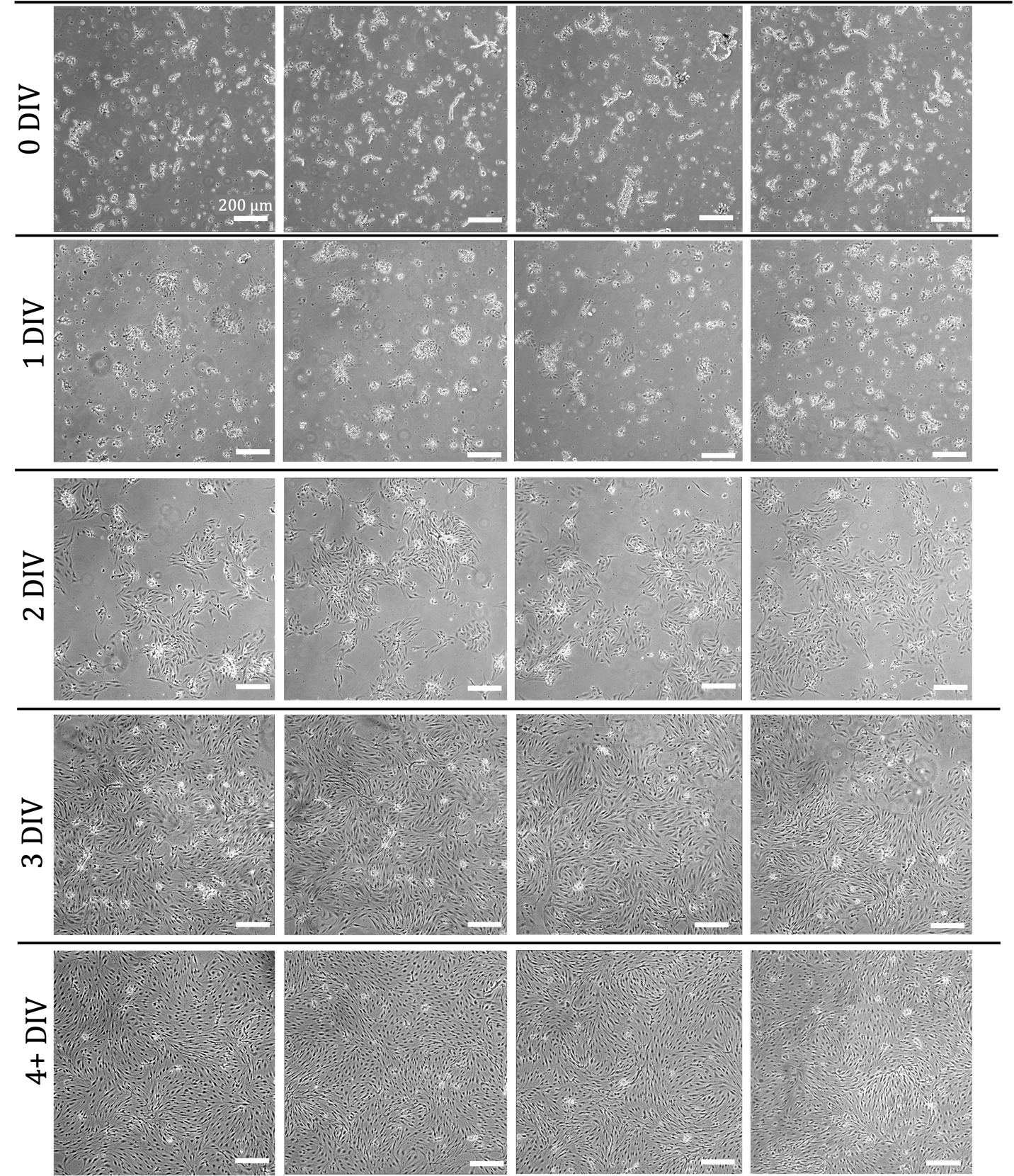
Supplementary Figure 6: Endothelial Cell Culture Growth:** These images document the range of morphology exhibited by primary isolated endothelial cell cultures. Each image was taken with phase contrast microscopy and scale bars are 200 μm.

**
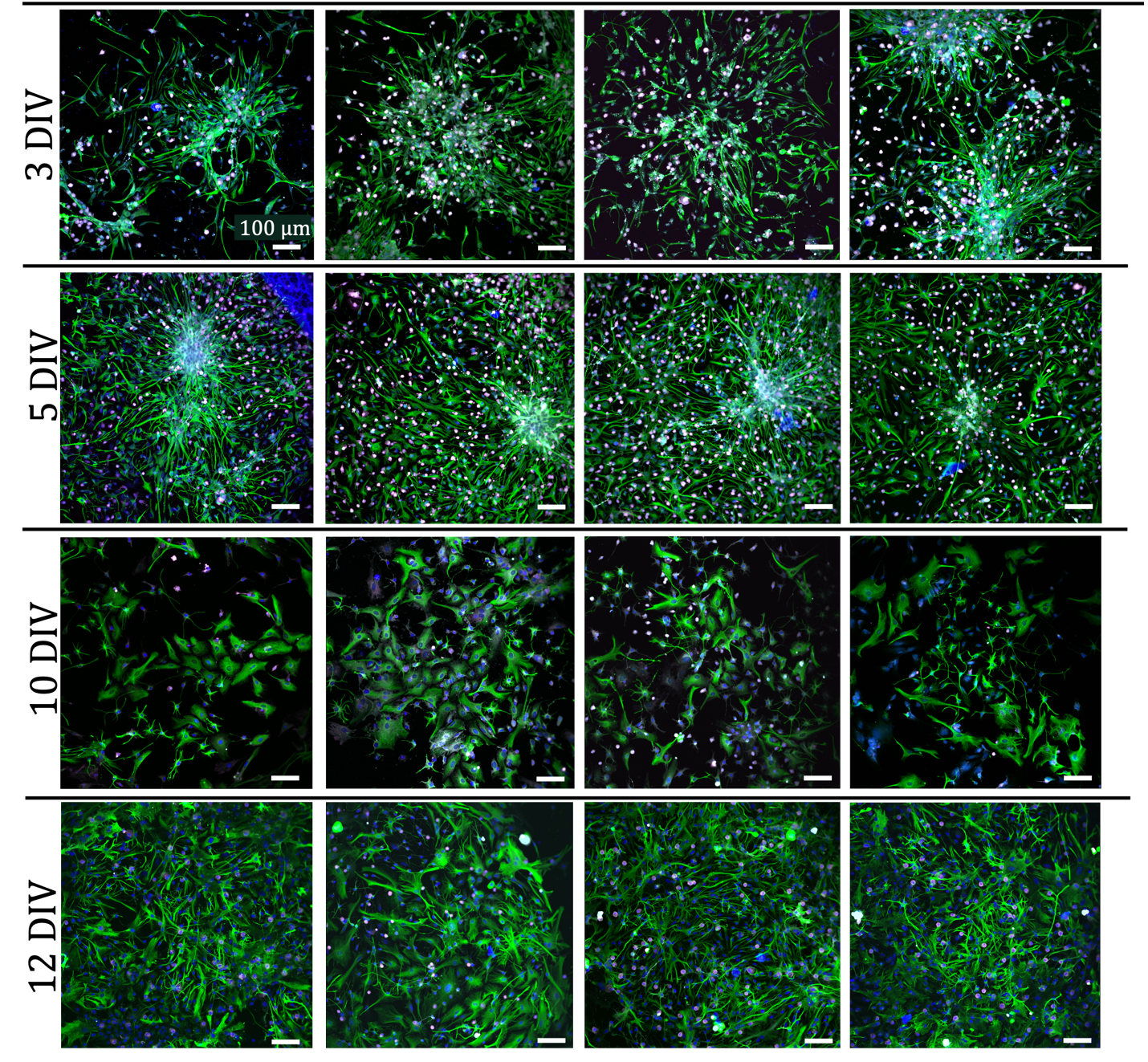
**

**Supplementary Figure 7: Mixed Glial Growth vis Immunostaining:** These images document the range of morphology and GFAP (green), MAP2 (light blue), and Iba1 (pink) expressed by primary isolated mixed glial cultures along with DAPI (blue) at four timepoints. Each image was taken with confocal microscopy and scale bars are 100 μm.

**ISOLATION SOLUTION PREPARATION PROTOCOLS**

Dissection media (vacuum filtered)

- 3.2 g glucose
- 500 ml HBSS (Ca and Mg Free)
- 1% penicillin/streptomycin

Enzymatic Dissociation Media 1 (syringe filtered) (per brain)

- 5 mL of DMEM
- 5 mL Collagenase Type II (1 mg/mL).

Enzymatic Dissociation Media 2 (syringe filtered) (per brain)

- 5 mL of DMEM
- 2.5 mL Collagenase Type II (0.5 mg/mL).
- 5 mL Collagenase/Dispase (2 mg/mL).

20% BSA Solution (syringe filtered) (per brain)

- 2 g Bovine Serum Albumin (BSA)
- 10 mL DMEM

33% Percoll Solution (syringe filtered) (per brain)

- 1 mL 10x PBS
- 9 mL Percoll
- 1mL 1x PBS
- 1 mL FBS

**CULTURE MEDIA PREPARATION PROTOCOLS**

Astrocyte Growth Media (vacuum filtered)

- DMEM high glucose
- 10% FBS
- 1% penicillin/streptomycin

Pericyte Growth Media (vacuum filtered)

- DMEM low glucose
- 10% FBS
- Gentamycin sulfate (50 ug/mL)

Endothelial Growth Media (vacuum filtered)

- DMEM/F12
- 10% BPDS
- Heparin (0.1 mg/mL)
- bFGF (5 ug/mL)
- Insulin-Transferrin-Sodium Selenite Supplement (50 ug/mL)
- Gentamycin sulfate (50 ug/mL)
- puromycin (5 ug/mL)
